# Prion protein N1 cleavage peptides stimulate microglial interaction with surrounding cells

**DOI:** 10.1038/s41598-020-63472-z

**Published:** 2020-04-20

**Authors:** J. A. Carroll, B. R. Groveman, K. Williams, R. Moore, B. Race, C. L. Haigh

**Affiliations:** Laboratory of Persistent Viral Diseases, National Institute of Allergy and Infectious Diseases, Division of Intramural Research, Rocky Mountain Laboratories, National Institutes of Health, 903 South 4th Street, Hamilton, MT 59840 USA

**Keywords:** Extracellular signalling molecules, Microglia

## Abstract

Microglia act as the protective immune cell of the brain. By surveying the tissue to identify and rectify problems, they function to maintain the health of brain cells. The prion protein N-terminal cleavage fragment, N1, has demonstrated neuroprotective activities *in vitro* and *in vivo*. This study aimed to elucidate whether N1 could modulate microglial function and, if so, determine the consequences for the surrounding tissue. Using a mixed neuronal lineage and microglia co-culture system, we showed that N1 stimulation changed overall morphology and metabolism, suggesting enhanced cellular viability. Furthermore, N1 induced an increase in Cxcl10 secretion in the co-cultures. Recombinant Cxcl10, administered exogenously, mediated the changes in the mixed neuronal lineage culture morphology and metabolism in the absence of microglia, but no effect of Cxcl10 was observed on microglia cultured on their own. Direct cell-to-cell contact was required for N1 to influence microglia in the co-cultures, and this was linked with restructuring of microglial membrane composition to include a higher GM1 content at interaction sites with surrounding cells. Our findings show that N1 can play a regulatory role in microglial function in the context of an inter-connected network of cells by changing both cellular interaction sites and cytokine secretion.

## Introduction

Within the brain different cell types act synergistically to maintain the function of the whole organ. Despite our understanding of the roles of different cellular types, there is still much we do not know about inter-cellular communications. Microglia (MG) have many essential functions in the healthy central nervous system that include neurodevelopment, synapse sensing and remodeling, and immune surveillance^1–4^. They are known to survey the tissue looking for infection or damage and react to remove or correct this accordingly^5,6^. Depending upon the nature of the stimulus, MG may react by assuming a phagocytic phenotype or by changing their cytokine secretion^2,7^. The signals that change the activation states of MG are diverse requiring complex cellular-crosstalk to communicate cell health^5,6,8^.

The prion protein (PrP) is a small, plasma membrane-tethered glycoprotein of unresolved function. In addition to glycosylation and membrane tethering via a glycosylphosphatidylinositol (GPI) anchor, PrP undergoes various other post-translational modifications. These post-translational modifications include endoproteolytic cleavage at three or more sites^9,10^. Of the cleavage events, termed α-, β- and γ-cleavages, the α- and β-cleavages have been most widely studied. The α-cleavage site is around amino acid 111 and produces N1 (~23–111) and C1 (~112–231) fragments with β-cleavage occurring around residue 89 and producing N2 (~23–89) and C2 (~90–231) fragments. The C-terminal cleavage fragments retain the GPI anchor and remain associated with cell membranes whereas the N-terminal cleavage fragments are no longer tethered and predominantly detected extracellularly in culture^11–13^.

Despite no consensus regarding the function of PrP, numerous studies have linked it with neuroprotection. Murine PrP knock-out models show only mild CNS phenotypes when unstressed^14,15^. However, when subjected to an insult, for example ischemic infarct, PrP knock-out mice suffer greater damage and when PrP is over-expressed damage is reduced compared with wild type mice^16,17^. Both the α- and β-cleavages have been linked with neuroprotection^18^ and the N1 and N2 N-terminal secretory fragments exhibit their own neuroprotective activities^11,19–21^. In monocultures, N1, N2 or both peptides have shown the capacity to modulate intracellular reactive oxygen species (ROS), signal transduction through MEK1 and p53, changes in plasma membrane lipids and mitochondrial fission^11,19,21,22^. Several of these factors are activators of MG^23–25^, therefore the N1 and N2 secretory fragments may have a direct influence on MG function. The N1 fragment has additionally been found to exert neuroprotective actions against the beta-amyloid peptides implicated in Alzheimer’s Disease pathogenesis. The neuroprotection conferred by N1 in AD is thought to be due to direct binding and this aspect of N1 biology is currently under investigation as a potential therapeutic avenue^26–29^.

With the increased interest in the neuroprotective qualities of N1, it remains prudent to fully understand the functional behaviors of this peptide. While the activity of the N1 peptide has been considered in the context of neuronal cell lines and neural stem cells (NSCs), these homogenous cultures show only individual cell type responses to N1 and do not represent the complexity of cellular interactions that occur within the brain. Further, the action of N1 on MG function has not yet been considered. To redress this, we generated a co-culture system to resemble the cellular cross-talk of the brain in which neurons, astrocytes and oligodendrocytes (neuronal lineage cells), and microglia were exposed to the N1 peptide. We queried whether the same cellular protective functions would be produced in MG within the co-cultures and, if so, what the consequences would be for the health of the culture. Our findings showed that N1 could exert modulatory effects on MG only in the context of a mixed culture of cells where MG could make direct contact with the surrounding cells.

## Methods

### Ethics statement

All mice were housed at the Rocky Mountain Laboratories (RML) in an AAALAC-accredited facility in compliance with guidelines provided by the Guide for the Care and Use of Laboratory Animals (Institute for Laboratory Animal Research Council). Experimentation followed RML Animal Care and Use Committee approved protocol 2016–058.

### NSC harvest, culture and differentiation

Neural stem cells (NSCs) were harvested from the brains of 8–12 week old C57B/10SnJ mice by coarse dissection of the sub-ventricular zone followed by mechanical and enzymatic dissociation of NSCs from the tissue as previously described^30^. Routine culture was carried out using Neurocult complete proliferation medium (StemCell Technologies) supplemented with 20 ng/ml EGF, 10 ng/ml FGF and 2 µg/ml heparin (StemCell Technologies, USA). Neurospheres were expanded using mechanical passaging, which has been described in detail previously^31^.

Differentiation was initiated by transferring NSCs into complete differentiation medium (StemCell Technologies). Neurospheres were dissociated into a single cell suspension and plated into poly-d-lysine coated chambered coverslips or well inserts at a concentration of 3.6 × 10^4^ cells/well and cultured for six days before introduction of MG. During co-culture all cells were maintained in the complete differentiation medium under normal incubator conditions (5% CO_2_, 37 °C, humidified).

### Microglia harvest, purification and culture

Cx3cr1 knockout mice homozygous for the Cx3cr1-GFP targeted mutation (B6.129P-Cx3cr1tm1Litt/J, The Jackson Laboratory, Stock No: 005582) were crossbred with Ccr2 knockout mice homozygous for the Ccr2-RFP targeted mutation (B6.129(Cg)-Ccr2tm2.1Ifc/J, The Jackson Laboratory, Stock No:017586) to generate transgenic mice expressing GFP in microglia and RFP in macrophage (TgGFP/RFP mice). Resultant offspring had heterozygous expression of both Cx3cr1 and Ccr2. Microglia were isolated from six to eight-week-old TgGFP/RFP or C57BL/10SnJ mice obtained from an in-house breeding colony. Mice were euthanized then transcardially perfused with 10 ml sterile phosphate-buffered saline (PBS, Gibco). Each brain was removed and diced into 2 mm pieces with a sterile scalpel and further dissociated using the Neural Tissue Dissociation Kit – Postnatal Neurons (catalog # 130-094-802) in combination with the gentle MACS Dissociator for automated tissue separation into a single-cell suspension (Miltenyi Biotec). Tissue dissociation was carried out per the manufacturer’s instructions.

The single-cell suspension for each brain was strained through a 70 μm MACS SmartStrainer (Miltenyi Biotec), centrifuged (300 × g, 23 °C, 10 minutes), and suspended in 1.8 ml sterile PBS, pH 7.2, supplemented with 0.5% bovine serum albumin (PBS-BSA). Myelin contamination was reduced by the addition of 200 µl Myelin Isolation Beads (Miltenyi, product # 130-104-253) to the cell suspension and incubated for 15 minutes at 4 °C. The volume was then adjusted to 3.0 ml with PBS-BSA and subjected to magnetic activated cell separation (MACS) by adding 1.0 ml to 3 LS Columns (Miltenyi Biotec). The flow-through was collected (unlabeled cell suspension), and each column was washed twice with 1.0 ml of PBS-BSA. All washes were then combined with the collected flow-through, yielding a total of approximately 9.0 ml of a myelin-reduced cell suspension for each brain. The myelin-reduced single-cell suspension was then centrifuged (300 × g, 23 °C, 10 minutes) and cells were suspended in 180 µl PBS-BSA for microglia isolation.

Microglia from a single mouse brain were labeled in the single-cell suspension by adding 20 µl CD11b MicroBeads (product # 130-093-634, Miltenyi Biotec) with incubation for 15 minutes at 4 °C. The volume was then increased to 5.0 ml with PBS-BSA and cells were pelleted by centrifugation (300 × g, 23 °C, 10 minutes). The washed cells were suspended in a final volume of 1.0 ml PBS-BSA, applied to a LS Column, and labeled microglia were isolated by MACS. The column was rinsed 3 times with 3.0 ml PBS-BSA, where the flow-through was discarded. The LS Column containing isolated microglia was removed from the magnetic field, 5.0 ml PBS-BSA was added to the column, and microglia were flushed into a fresh sterile tube using the supplied plunger. We typically acquire approximately 4 × 10^5^ microglia per adult mouse brain using this method of isolation, with ≥99.9% purity (as shown in Supplementary Figure S1).

Purified microglia were centrifuged (300 × g, 23 °C, 10 minutes) and suspended in DMEM/F-12 Glutamax (Invitrogen) supplemented with 10% FCS, 100 U/ml penicillin, 100 ng/ml streptomycin, 10 ng/ml mouse recombinant carrier free MCSF (CSF-1, R&D Systems), and 50 ng/ml human recombinant TGFβ1 (Miltenyi Biotec)^32^. Microglia were then transferred into 25 cm^2^ Ultra-Low Attachment polystyrene flasks (Corning) and incubated at 37 °C with 5.0% CO_2_. The medium was changed every 5 days with fresh medium. Microglia were routinely cultured between 10 and 15 days before harvesting and adding to differentiated mixed neuronal-lineage cultures.

At the time of starting the co-cultures, microglia were aspirated from the flasks, which were then washed briefly with accutase and the accutase wash combined with the rest of the microglia. Cells were collected by centrifugation (300 × g, 5 minutes), then counted using the Muse Count and Viability Kit (Millipore-Sigma) with the Muse flow cytometer (Millipore-Sigma). Five thousand live cells were plated into the co-cultures or into their own wells for monocultures.

### Peptides

Peptides were purchased from China Peptide (China) and Synpeptide (China). Both batches were purified to >95% purity by HPLC and validated by mass spectrometry (See Supplementary Data S2 for Synpeptide quality control data). No variation in peptide activity was observed between batches. Each batch was reconstituted in water and the concentration adjusted to 100 µM based on spectroscopy using 41480 (N1) and 35980 (N2) extinction co-efficients. To block the basic charged residues of N1, sulfo-NHS acetate (Thermo Scientific) was reacted with primary amines by incubation of equal masses of peptide and sulfo-NHS acetate in PBS for 1 hour at room temp. Reacted peptides were washed 3 × in 3 kDa cut-off spin columns in PBS, then resuspended in PBS to a final concentration of 100 µM. This reaction produced extensive modification of the peptide (Supplementary Figure S3).

### Digestion and mass spectrometry analysis of N1 and Acyl-N1 peptide fragments

To assess the level of modification of the Acyl-N1 peptide, 2.0 µg of N1 or Acyl-N1 peptides was suspended in 100 mM Tris-HCl, 10 mM CaCl_2_ and digested with 2.0 µg Chymotrypsin (sequencing grade, Promega) for 17 hours at 24 °C. The digestion was halted by the addition of trifluoroacetic acid (TFA) to a final of 0.5%. The reaction was desalted using Pierce peptide desalting spin columns (Thermo Scientific) per the manufacture’s instruction. Peptides were eluted from the columns by applying 300 µl of a 60:40 mixture of acetonitrile (ACN):ultra-pure H_2_O, 0.1% TFA (2 times). The eluates for N1 or acyl-N1 were collected and dried using a CentriVap Speed Vacuum. Eluted peptides were then reconstituted in 3.0% ACN, 97% ultra-pure water, 0.1% formic acid (FA) to an estimated concentration of 1 pmol/µl for mass spectrometry analysis.

The digested N1 and Acyl-N1 peptides were analyzed by LC–MS/MS using an Agilent 1290 UHPLC with a Dual AJS source coupled to an Agilent 6550 iFunnel Quadrupole Time-of-Flight (Q-TOF) system (Agilent Technologies). Progressively increasing injections ranging from 0.1 µl to 2.0 µl were loaded onto a 40 nl trapping column using Buffer A (0.1% FA in H2O) and then separated on a 2.1 × 50 mm column (Agilent InfinityLab Poroshell 120 EC-C18). Separation was achieved with a reversed phase step gradient at 250 μl/minute starting with 100% Buffer A to 30% Buffer B (90% ACN, 0.1% FA) in 20 min, then to 60% Buffer B in 24 min, and then held at 95% Buffer B for 3 min and back to 100% Buffer A by 29 min. The survey scan was done with an m/z range from 300 to 1600 at a scan rate of 3 spectra/second and a maximum of 3 precursor ions selected per cycle. The MS/MS scan rate was 2 spectra/second with an m/z range from 80 to 1600. Raw data files were searched using PEAKS Studio X build 20190129. Data were processed using a parent mass error tolerance of 20 ppm and a fragment mass error of 0.2 Da. Data were searched with variable modifications of +16 for methionine oxidation and 42.01 for acetylation at lysine using a fasta database consisting of mouse Prion Protein, bovine chymotrypsin and various common contaminants.

### Cxcl10 and Cxcr3 blocking

Recombinant mouse Cxcl10 was purchased at >97% purity, <0.01 EU per µg endotoxin, Effective Dose 0.05–0.3 µg /mg (R&D Biosystems) and was reconstituted in sterile phosphate buffered saline (PBS) containing 0.1% bovine serum albumin to a final concentration of 100 µg/ml. Cxcl10 solution was aliquoted and stored at −20 °C until use. The Cxcr3 neutralizing antibody was purchased from Abcam (ab64714) and included in cell medium at a concentration of 5 µg/ml.

### Mixed ganglioside solution

A ganglioside mixture containing GM1, GD1a, GD1b and GT1b derived from bovine brain (Calbiochem) was reconstituted in ethanol:methanol:water (2:1:0.1 v/v) to a concentration of 10 mg/ml. This was diluted into normal media to a final assay concentration of 50 µg/ml.

### Prestoblue cell viability/metabolism assay

Cells were incubated in a 1 in 10 dilution of Prestoblue metabolism reagent (Invitrogen) in normal medium, for 30 minutes under standard incubator conditions. Three replicate aliquots of 100 µl media per well per independent repeat were transferred into a black 96 well plate and read in a BMG ClarioStar (BMG Labtech) using 535 and 615 nm excitation and emission wavelengths respectively.

### Cytokine and Chemokine quantification

Levels of 33 mouse cytokines and chemokines were analyzed using the Bio-Plex Pro™ Mouse Chemokine Panel 33-Plex (product #12002231, Bio-Rad) in combination with a Bio-Plex 200 Suspension Array System. This panel measures: BCA-1/Cxcl13, CTACK/Ccl27, ENA-79/Cxcl5, Eotaxin/Ccl11, Eotaxin2/Ccl24, Fractalkine/Cx3cl1, GM-Csf /Csf-2, I-309/Ccl1, I-TAC/Cxcl11, IFN-γ, IL-1β, IL-2, IL-4, IL-6, IL-10, IL-16, IP-10/Cxcl10, KC/Cxcl1, MCP-1/Ccl2, MCP-3/Ccl7, MCP-5/Ccl12, MDC/Ccl22, MIP-1α/Ccl3, MIP-1β/Ccl4, MIP-2/Cxcl2, MIP-3α/Ccl20, MIP-3β/Ccl19, RANTES/Ccl5, SCYB16/Cxcl16, SDF-1α/Cxcl12, TARC/Ccl17, TECK/Ccl25, and TNF-α. Assays were performed using 50 µl of undiluted culture medium per sample.

### Mitotracker staining and live cell imaging

MitoTracker™ Green FM (Invitrogen) was dissolved in anhydrous DMSO to a final concentration of 1 mM. Cells were labelled using a final concentration of 100 nM MitoTracker in normal medium for 20 minutes. Medium was changed before imaging. For low magnification analysis of culture area, images of the entire well were captures using a well-scanning protocol on an EVOS (Invitrogen) widefield microscope system with a GFP (470/22 nm excitation, 510/42 nm emission) light cube. Image analysis was carried out by subtraction of non-tissue background pixels (thresholds set identically for all images) and calculation of the total slide area covered by all positive pixels achieved using Fiji imaging software^33^.

### GFP live cell imaging and morphology analysis

GFP was imaged in live cultures using an EVOS (Invitrogen) widefield microscope system with a GFP (470/22 nm excitation, 510/42 nm emission) light cube. Microglia were considered ‘ramified’ if they showed a generally smaller cell body with branching processes extended outward. ‘Non-ramified’ encompassed cells with larger cells bodies, no or few processes, or those with a more poached egg appearance. Between 4 and 10 fields of 10–30 cells were counted per independent repeat.

### MitoSOX staining and live cell imaging

MitoSOX™ (Invitrogen) stock solutions were prepared by dissolving probe in anhydrous DMSO to a final concentration of 5 mM. Working solutions were prepared in normal medium to a final concentration of 5 µM and cells were labelled for 10 minutes before imaging in fresh medium. Live cell images were collected using a Nikon A1 fluorescence microscope.

### Dichlorofluorescein diacetate (DCFDA) assay

The DCFDA assay has been described in detail previously^31^. Briefly, cells were loaded with CM-H_2_-DCFDA by incubating in a 5 µM in PBS working solution for 20 minutes at 30 °C in the dark. Cells were transferred into phenol-red-free OptiMEM (Invitrogen) and readings were taken every 5 minutes using 488 nm and 530 nm excitation and emission wavelengths in a BMG ClarioStar.

### GM1 staining

AlexaFluor-647 cholera toxin B (CTx-B) conjugate (Invitrogen) was diluted to 1 mg/mL in PBS for immediate use. Cells were labelled with a final concentration of 3 µg/mL in normal media for 5 minutes at 37 °C in the dark. Fluorescent images of live cells were captured immediately using a Nikon A1 fluorescence microscope. AlexaFluor-647 cholera toxin B conjugate intensity was calculated using microglial GFP to draw an outline around each individual cell then AlexaFluor-647 pixel intensity per cell was measured. The same measurement protocol was used for the sites of direct cell-cell interaction with the cell body excluded from the analysis. Between 4 and 10 fields of 10–30 cells were counted per independent repeat.

### Immunofluorescence

Immunofluorescent staining was carried out following washing in PBS and fixing in 4% (v/v) paraformaldehyde. Cells were permeabilized for 10 minutes in 0.01% (v/v) triton-X-100, then blocked with 10% (v/v) FBS and 0.1% (w/v) bovine serum albumin (BSA) in PBS for 30 minutes before being transferred in to primary antibody in a 1% (v/v) FBS and 0.1% (w/v) BSA in PBS solution overnight at 4 °C. The primary antibodies used were NF-L (Invitrogen), GFAP (Abcam) and OSP (Abcam) at a 1 in 50 dilution. Cells were washed 3× in PBS before addition of AlexFluor-488 or −647 secondary antibodies at a 1 in 250 dilution and incubated for 2 hours at room temperature in the dark. Cells were washed again 3× in PBS then mounted in Fluoromount with DAPI and allowed to cure for 24 hours before imaging. Images were collected using a Nikon A1 fluorescence microscope. For analysis of cell contact, microglia were considered ‘MNLC associated’ if they were directly contacting a neighboring cell, cells that were spatially isolated in the well were classified as ‘unassociated’.

### Bead phagocytosis assay

Bead phagocytosis assays were carried out as described in Lian *et al*.^34^. Briefly, green fluorescent latex beads were opsonized in FBS for 1 h at 37 °C at a ratio of 1:5 beads to FBS. This solution was then diluted into complete differentiation medium to a final concentration of 0.01% (v/v) beads. MG were treated with PBS (control), 1 µM N1 or 10 µM tyrphostin-A23 (Sigma-Aldrich) for 1 hour before exchanging the media for the bead-containing media. MG were incubated for a further hour with the beads before washing with cold PBS and fixing in 4% (v/v) paraformaldehyde. The number of cells showing uptake of the fluorescent beads was counted in three or more fields of 50–100 cells per independent repeat.

### Statistical analysis

Statistical analyses were carried out using Graphpad Prism 6. Two-way ANOVA with Bonferroni’s secondary testing was done for analyses of two or more groups, otherwise one-way ANOVA with Tukey’s secondary testing was applied. All graphs show the mean and s.e.m. of ‘*n*’ independent repeats and the numerical data contained within the plots can be found in Supplementary Data S4. A *p*-value of ≤0.05 considered significant.

## Results

### Co-culture design

**Figure 1:**
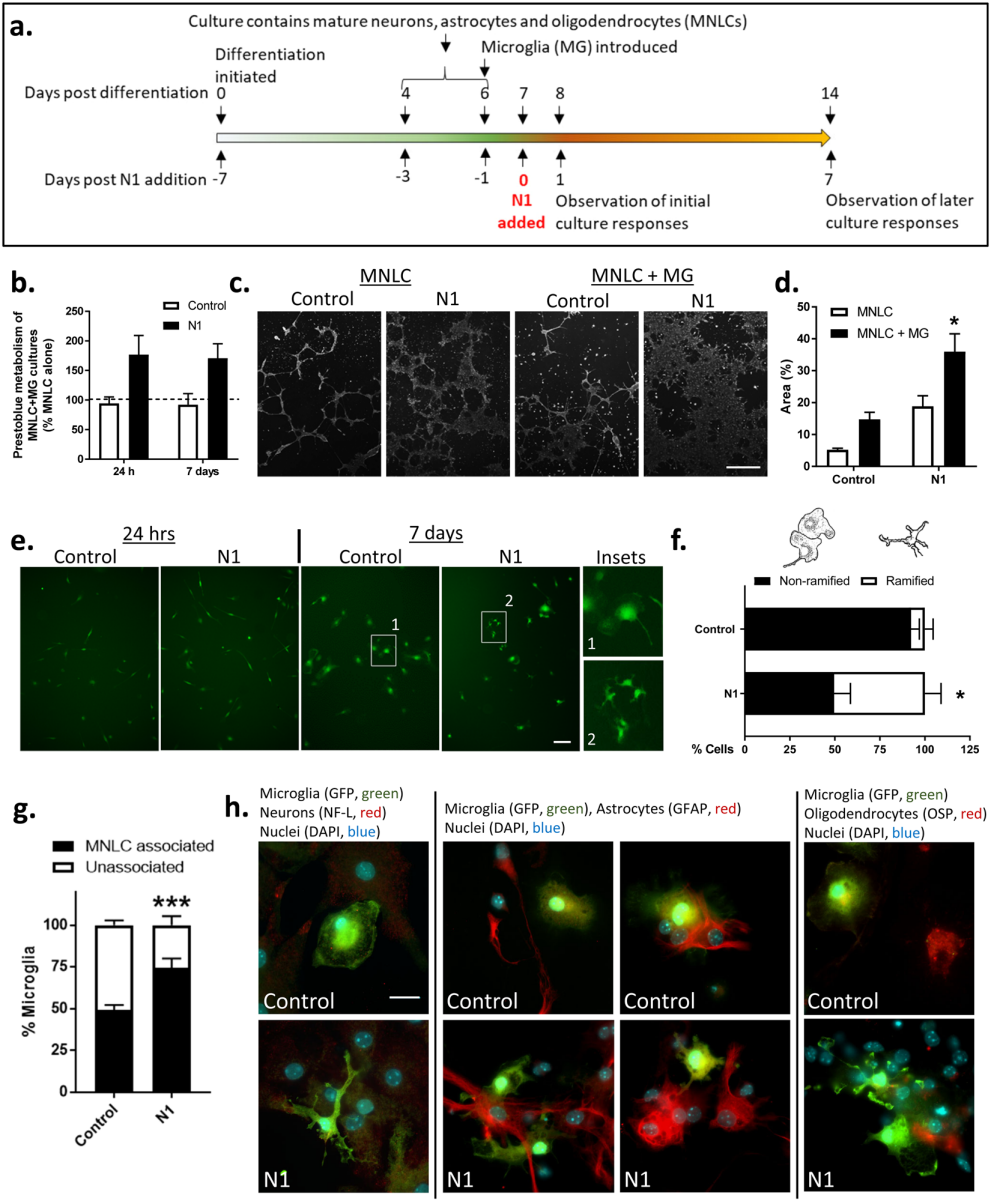
N1 addition to MNLC-MG co-cultures influences whole culture and MG morphology after 7 days. (**a**) Timeline schematic showing differentiation of the MNLCs, MG introduction, N1 addition and experimental observation points. (**b**) Prestoblue metabolism of the N1or PBS treated MNLC-MG co-cultures shown as a percentage of the MNLC alone responses (dotted line) at 24 hours and 7 days. *n* = 4. (**c**) Low magnification (10×), live cell MitoTracker-green staining after 7 days with or without N1. Scale bar = 50 µm. (**d**) Quantification of the surface area covered by the cultures after 7 days co-culture. *n* = 3. (**e**) Live imaging of MG morphology at 24 hours and 7 days after addition of N1. Scale bar = 20 µm. (**f**) Count of the number of microglia displaying ramified morphology under control and N1 treated conditions after 7 days. Cell sketches shown above the graph represent examples of MG classified as non-ramified vs ramified. *n* = 3. (**g**) Count of the number of microglia associated with MNLC tissue after 7 days. *n* = 3. (**h**) MG morphology and association with different cell types following fixing and staining. Scale bar = 15 µm. Further NF-L staining and no primary background control staining is shown in Sup Fig. 1. Graphs show the mean and s.e.m. of ‘*n*’ independent repeats. *p < 0.05, ***p < 0.001.

We devised a co-culture system to investigate the influence of the PrP N-terminal cleavage fragment, N1, on microglia function and the consequences for other brain cell types. NSC cultures were used to differentiate mature cultures containing neurons, astrocytes and oligodendrocytes. Differentiation of NSCs results in mature cells appearing in 4–6 days^35,36^ with progenitors negligible (<1%) by 5 days^36^ and markers of mature cells are strongly detected by 7 days within these cultures^31^. The mature cultures do not differentiate equal populations but contain ~80% astrocytes, <20% neurons and <5% oligodendrocytes^37–39^. The cultures will be referred to hereafter as mixed neuronal-lineage cultures (MNLCs). From our previous model development we know that MG will integrate into the MNLCs^40^, therefore we set up a monolayer co-culture system. The NSCs were differentiated for 6 days into MNLCs with MGs added into the culture on day 6. The co-culture was incubated together for a further 24 hours before starting experiments to allow the MG to integrate. A schematic of the experimental timeline is shown in Fig. 1a.

### N1 increases metabolism and changes culture morphology in MNLC-MG co-cultures

Previous studies have found that N1, administered at a concentration of 1 µM, was able to stimulate neuroprotective signaling and influence neural stem cell growth^19,41^. Therefore, at the start of the experiment 1 µM N1 (or the equivalent volume of PBS [control]) was included in the culture medium. The health of the co-culture was monitored after 24 hours and 7 days using Prestoblue cell viability reagent, which measures cell metabolism (Fig. 1b). Metabolism was increased in the MNLC-MG co-cultures that received N1. The increase in metabolism was maintained at 7 days indicating that the N1-MNLC-MG interaction was sustained and not toxic. To look more closely at the changed metabolism, mitochondria were stained with Mitotracker-green at 7 days post N1 addition. No changes in mitochondrial morphology were observed (data not shown). However, viewing the slides under low magnification revealed that the culture morphology was different when the MNLC-MG cultures received N1 (Fig. 1c). N1 treated co-cultures showed greater surface area staining with less tightly packed clusters of cells (Fig. 1d). While N1 on the MNLC alone and the control MNLC-MG cultures also indicated a modest increase over the MNLCs that received only PBS, the changes were not statistically significant. This suggested that the N1 and the MG together were synergistically acting on the cultures.

### N1 changes MG morphology in co-culture and enhances interactions with surrounding cells

To examine the morphology of the integrated MG, and to ensure that the greater viability/metabolism seen in the N1 co-cultures was not due to MG proliferation overwhelming the culture, the experiment was repeated using MG driving GFP off the *Cx3cr1* promoter. In this way GFP expressing MG could be distinguished from the MNLC cells. After 24 hours of N1 exposure the MGs did not look morphologically different to those seen in the control cultures (Fig. 1e) and no evidence of proliferation to produce the enhanced viability was observed. By 7 days, the GFP-expressing cells exhibited a different morphology within the cultures that received the N1 peptide when compared with controls. This morphological change, representing a more ramified appearance, was counted in approximately 50% of MG within the N1 treated cultures, compared with ~10% in the control cultures (Fig. 1f). To more closely observe the MG morphology and cell-cell interactions, 7-day post-treatment cultures were fixed and stained for markers of neurons (NF-L), astrocytes (GFAP), and oligodendrocytes (OSP). On initial observation, it was evident that less MG were associated with the MNLC cells in the cultures that did not receive N1. A count of MG that were associated with (directly contacting surrounding cells) or isolated from the rest of the culture confirmed significantly more cells were integrated in cultures that received N1 (Fig. 1g). When considering cell associations, neither the MG in the control nor the N1 treated cultures associated predominantly with cells positive for neuronal staining; only background fluorescence was visible around MG when staining with neurofilament-L (NF-L) (actual NF-L staining is shown in Supplementary Figure S5 for comparison). Instead, most MG interactions were with astrocytes with some localization adjacent to oligodendrocytes. This was true of both the control and N1 treated cultures but more distinct in the N1 treated cultures due to the heightened association (Fig. 1h).

### N1 has no detectible effects on MG metabolism, morphology or phagocytic activity in monoculture

**Figure 2:**
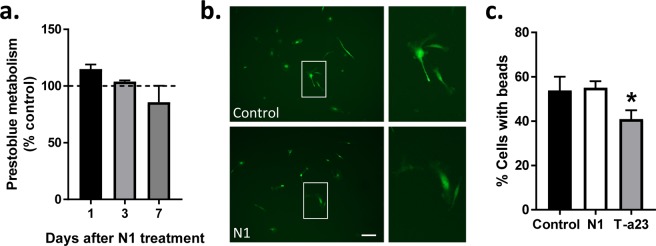
N1 does not influence MG metabolism, morphology or phagocytic function when grown as a monoculture. (**a**) Prestoblue metabolism of MG cultures following N1 exposure expressed relative to PBS controls (dotted line). *n* = 4. (**b**) Morphology of live MG 7 days following N1 addition. Scale bar = 20 µm. (**c)** Bead phagocytosis by MG one hour after exposure to N1 or tyrphostin-A23 (T-a23) internalization inhibitor. *n* = 3. Graphs show the mean and s.e.m. of ‘*n*’ independent repeats. *p < 0.05.

The N1 stimulated changes in MG morphology might be related to the increased cellular association. Therefore, we considered the responses of the MG to N1 when cultured alone. When MG were treated with N1 no change in their metabolism was seen up to 7 days (Fig. 2a) and no changes in morphology were manifest (Fig. 2b). Another indicator of changed MG function is phagocytosis. We additionally considered whether N1 may change MG phagocytic activity using a bead up-take assay (Fig. 2c). No change in phagocytosis from the control cells was observed. In the absence of other brain cell types, as provided by the MNLCs, N1 appears to have little or no detectable effect on MG.

### N1 stimulates increased Cxcl10 secretion in co-culture

**Figure 3:**
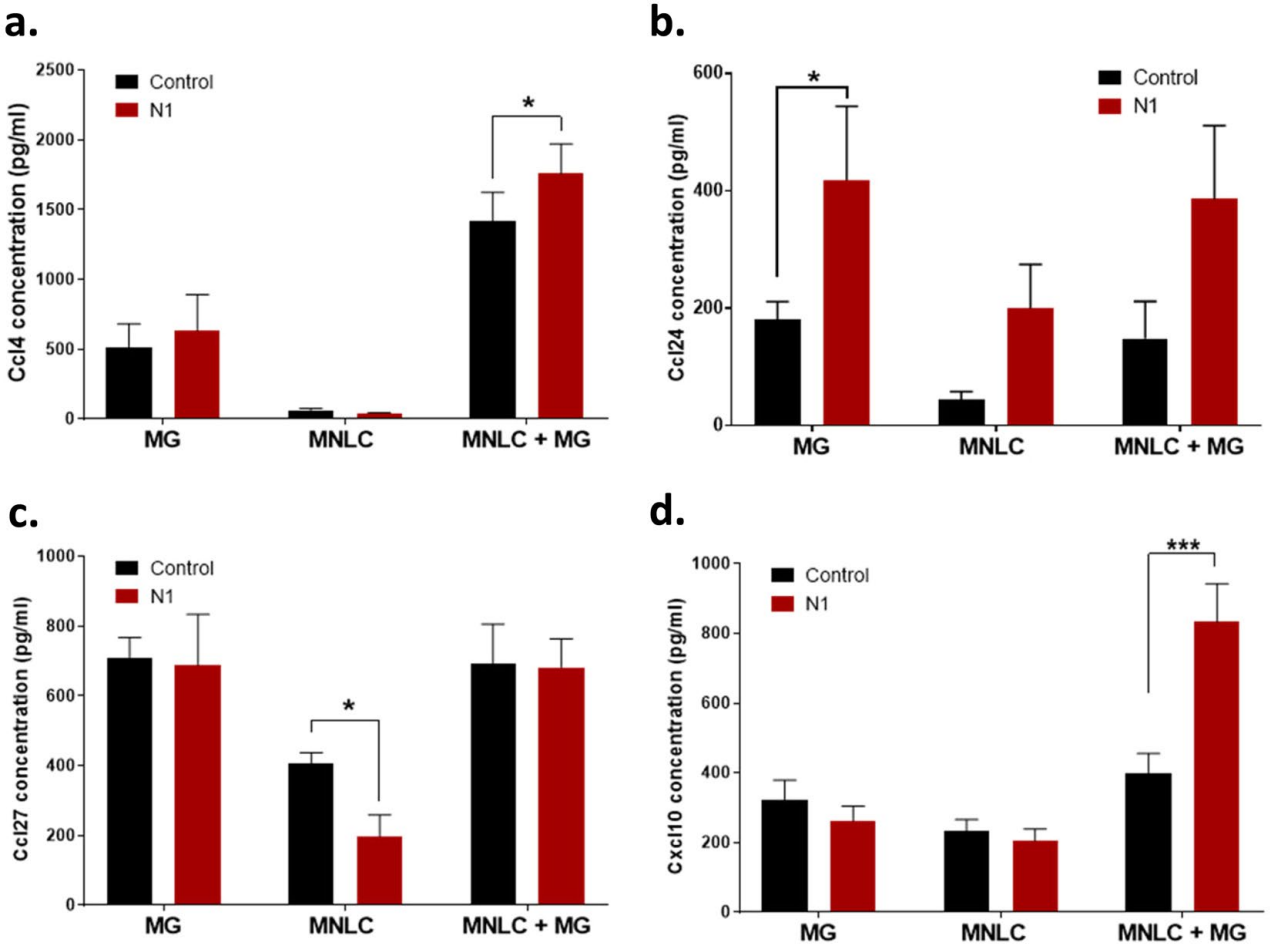
N1 mediated cytokine secretion changes. Concentration of (**a**) Ccl4, (**b**) Ccl24, (**c**) Ccl27, and (**d**) Cxcl10 in media from MG, MNLC and co-cultures with and without exposure to N1 for 24 hours. *n* = 4. Graphs show the mean and s.e.m. of ‘*n*’ independent repeats. *p < 0.05, ***p < 0.001.

MG activation results in different phenotypes in different contexts. Changes in MG activation may stimulate release of cytokines. To investigate how N1 was influencing MG function in the co-cultures, media samples were collected at 24 hours from the co-cultures, MNLCs alone and MG cultures alone for cytokine analysis. A panel of 33 pro-inflammatory cytokines were screened for changes; of these, only four demonstrated N1-related changes (Fig. 3; the full dataset is shown in Supplementary Table S6). Ccl4 showed an increase in the MNLC-MG co-cultures that received N1 (Fig. 3a), however, this was less pronounced than the increase induced by co-culturing suggesting that the co-culture environment is primarily responsible for this release. Ccl24 was significantly increased in the MG alone cultures but showed fluctuations in all N1 conditions (Fig. 3b). Ccl27 was decreased in the MNLCs treated with N1 alone but unchanged when MG are present (Fig. 3c). Cxcl10 showed a co-culture-N1-specific increase in secretion (Fig. 3d). Therefore, of the four cytokines showing N1 related changes in secretion, Cxcl10 appeared most specific to the co-cultures.

### Cxcl10 stimulates changed metabolism and culture morphology of NMLCs

**Figure 4:**
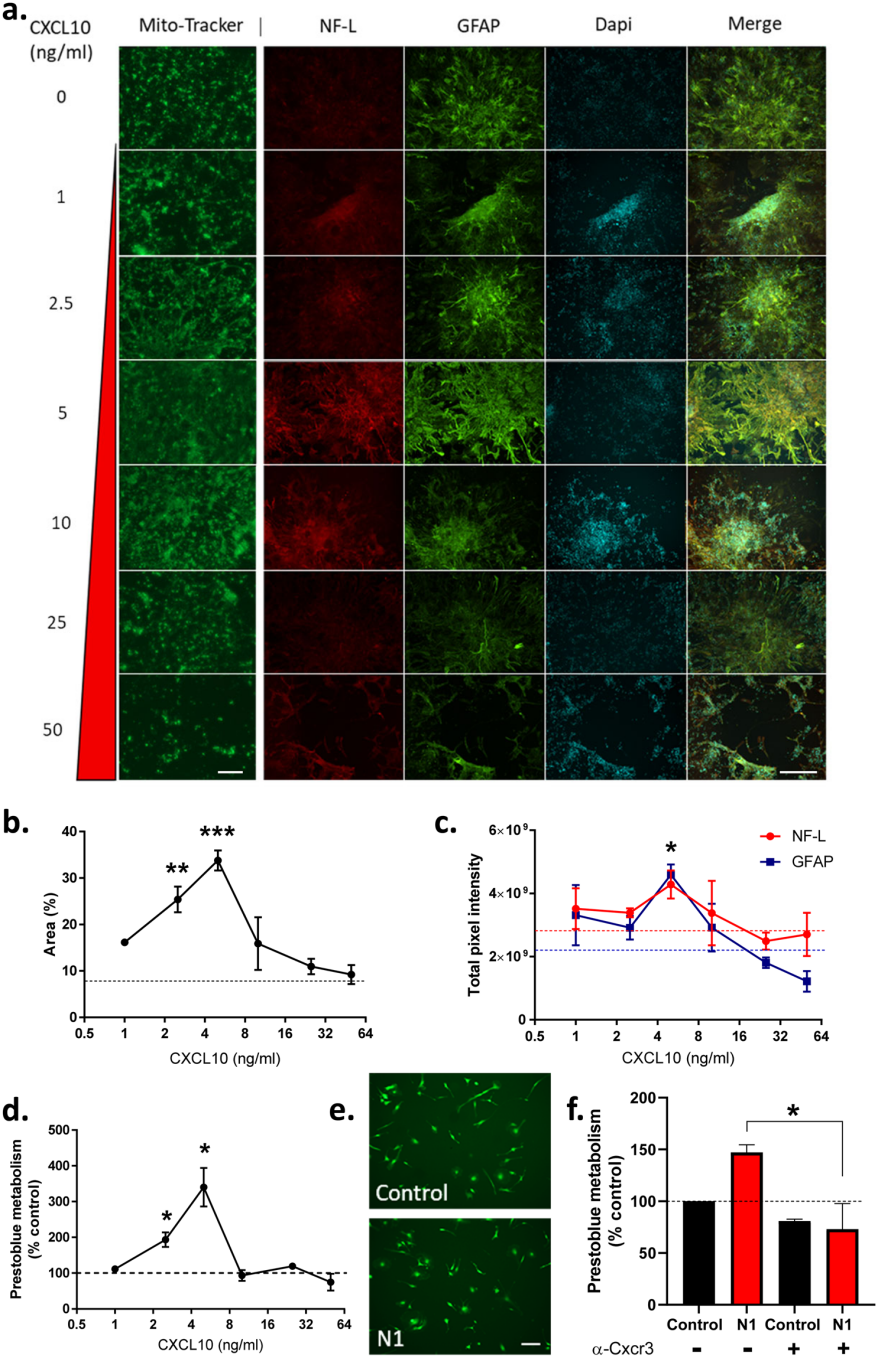
Cxcl10 influences MNLC morphology and metabolism. (**a**) Morphology of Cxcl10 treated cultures showing mitotracker staining in live cultures and neurons (NF-L) and astrocytes (GFAP) stained after fixation. Scale bars = 50 µm (mitotracker), 100 µm (NF-L/GFAP/DAPI/MERGE). (**b**) Area of cell coverage following Cxcl10 treatment. Dotted line denotes PBS control area coverage. *n* = 4. (**c**) Pixel intensity of neuronal and astrocytic staining. Dotted lines indicate untreated control staining for NF-L (red) and GFAP (blue) respectively. *n* = 3. (**d**) Prestoblue metabolism of Cxcl10 treated cultures relative to PBS controls. *n* = 4. (**e**) Morphology of live MG monocultures after 7 days treatment with 5 ng/ml Cxcl10. Scale bar = 20 µm. (**f**) Prestoblue metabolism of MNLC-MG co-cultures treated for 24 hours with PBS or N1 in the presence or absence of a Cxcr3 neutralizing antibody. *n* = 3. Graphs show the mean and s.e.m. of ‘*n*’ independent repeats. *p < 0.05. **p < 0.01, ***p < 0.001.

We queried whether Cxcl10 might be responsible for any of the changes seen in either the MNLCs or MGs. Seven-day-old MNLCs (the same age as for the beginning of N1 treatment) were treated with a range of doses of recombinant Cxcl10 and incubated for 7 days with a repeat dosage on day 3. The concentration curve was calculated based upon the measured N1 co-culture levels at 24 hrs (Fig. 3d) and attempted to account for both: 1. that the measured Cxcl10 in the media may be an underestimation as we could not measure exact consumption of the Cxcl10 by the cells, and 2. how much might be secreted over 7 days in co-culture. At 7 days Mitotracker-green staining showed that, with 2.5 and 5 ng/ml Cxcl10 administration, the MNLC morphology had changed. In similarity with the changes observed when the co-cultures were treated with N1, the Cxcl10 treated MNLCs covered a greater surface area of the slide (Fig. 4a,b). At higher concentrations this effect declined. Fixing the Cxcl10 treated MNLCs and staining for neurons and astrocytes showed that detection of both was higher at 5 ng/ml (Fig. 4a,c) suggesting enhanced survival of these cells. Likewise, measurement of Prestoblue metabolism showed a peak increase in culture metabolism at 5 ng/ml but this declined steeply afterwards (Fig. 4d). When MG were treated as monocultures with 5 ng/ml Cxcl10, the concentration that produced the optimal effect in the MNLCs, no difference in their morphology was observed after 7 days (Fig. 4e) and no changes in metabolism were measured (data not shown). To assess whether blocking the action of Cxcl10 would influence the stimulation of metabolism by N1, the co-cultures were incubated with a Cxcr3 (the primary Cxcl10 receptor) neutralizing antibody for one hour prior and for 24 hours of culture with the N1 peptide before assessing their metabolism. The cultures receiving the neutralizing antibody no longer showed a change in metabolism in response to the N1 peptide, indicating Cxcl10 may be mediating this change (Fig. 4f). Overall, Cxcl10 appears to mediate morphological and metabolism changes within the MNLC but demonstrates no influence on MG alone.

### A shared media environment is insufficient for N1 to stimulate changes in MNLC or MG cultures

**Figure 5:**
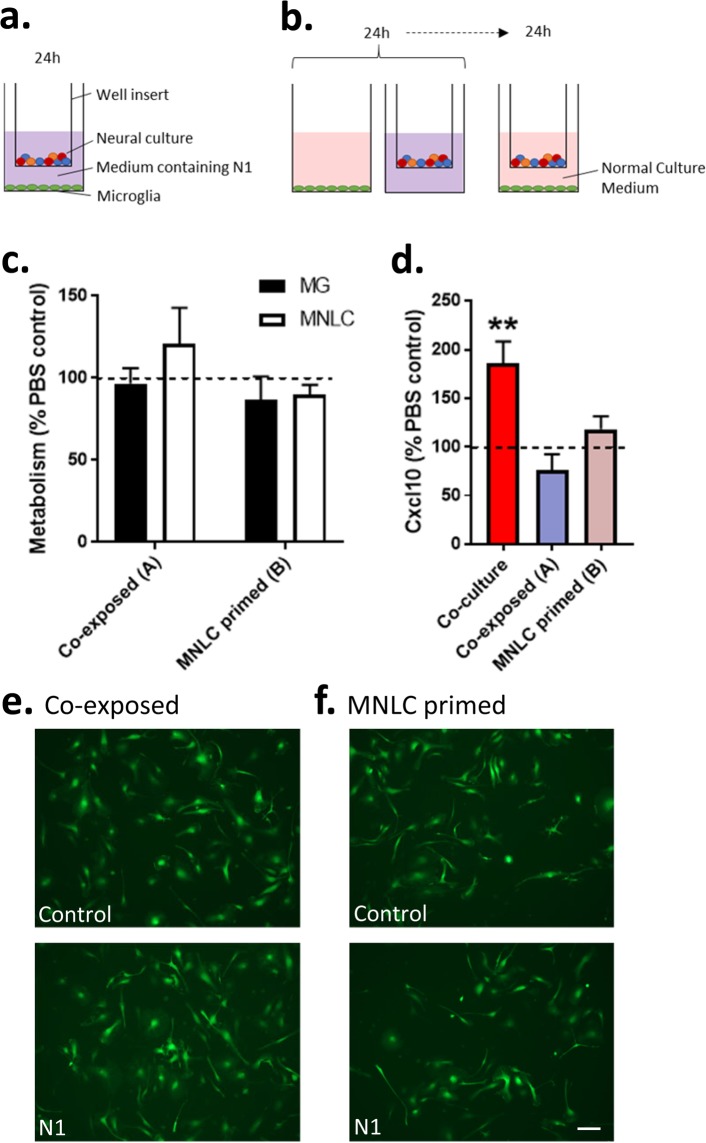
N1 mediated culture changes require direct cell contact. (**a,b**) Schematic of well insert experiments where (**a**) MNLC and MG are co-exposed to N1 for 24 hours and (**b**) MNLC are primed by exposure to N1 for 24 hours prior to co-culture. (**c**) Prestoblue metabolism after 24 hours co-incubation of the MG and MNLC co-exposed N1 and MNLC primed shown relative to control cells (dotted line). *n* = 3. (**d**) Cxcl10 detection in the media of the co-exposed and primed cultures, shown as a percentage of the PBS control medium (dotted line) and with the co-culture (MG integrated) results for comparison. *n* = 4. (**e,f**) Morphology of live MG at 7 days following the co-exposure (**e**) or priming of the MNLC (**f**). Scale bar = 20 µm. Graphs show the mean and s.e.m. of ‘*n*’ independent repeats **p < 0.01.

With exogenous Cxcl10 demonstrating the ability to produce similar changes within the MNLCs as MG co-culture with N1 induced, we next questioned whether the MG and MNLC cells needed to be in direct contact for N1 to mediate its effect or if cell communication through secretory factors was sufficient. To address this MNLCs were cultured in well-inserts with MG cultured within wells. In this way the cells could share the same media without directly contacting each other. Two experimental approaches were used; first, co-exposure of both the MNLC and MG to N1 at the same time (Fig. 5a). Second, to determine if factors secreted from the MNLC were influencing the MG, priming of the MNLC with N1 for 24 hours before transferring the insert into the shared MG well in the absence of N1 (Fig. 5b). Following 24 hours of co-incubation cells were assessed for metabolism and changes in Cxcl10 secretion. As the well inserts allowed for the cells to be separated, the metabolism of the MNLCs and MG was measured separately. This shows that neither experimental approach influenced the metabolism of either the MNLCs or the MG (Fig. 5c). Measurement of Cxcl10 in the media after 24 hours co-incubation showed no change in secretion regardless of co-exposure or priming (Fig. 5d). Using the *Cx3cr1* driven GFP to monitor the MG morphology after 7 days co-incubation revealed no changes in morphology (Fig. 5e,f). Together, these data suggest that direct cell-to-cell contact is required for N1 to influence both MNLC and MG.

### N1 does not influence redox balance in the MNLC-MG co-cultures

**Figure 6:**
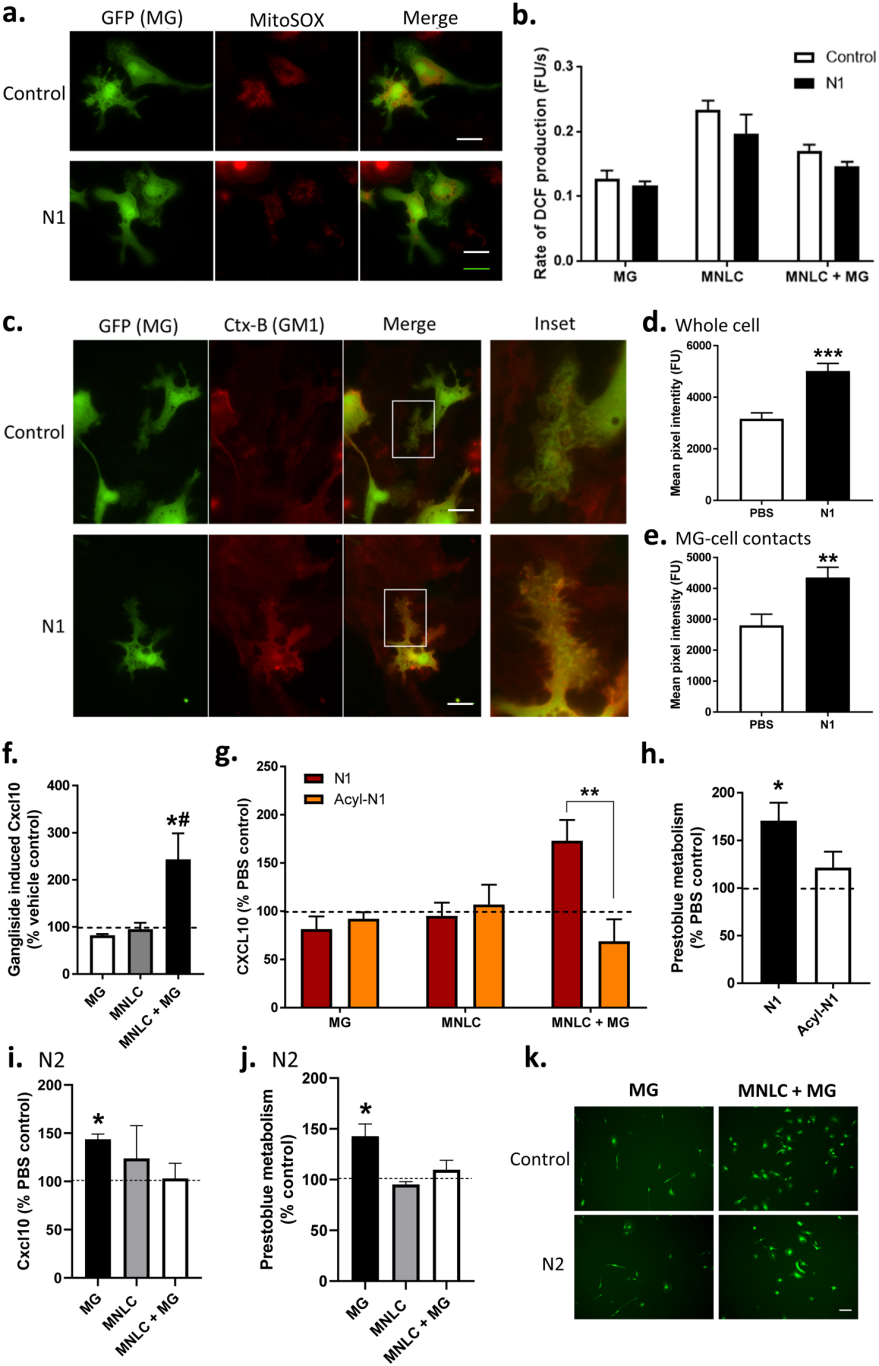
N1 does not change cellular ROS but mediates its effect through membrane changes at the site of MG-MNLC contact and its own charge. (**a**) MitoSOX live cell staining of mitochondrial superoxide production within MG in co-culture. Scale bars = 10 µm. (**b**) DCFDA assay for ROS production in MG, MNLC and MNLC-MG co-cultures treated with N1. *n* = 3. (**c**) CTx-B staining and live cell imaging of GM1 in the membranes of MG with and without N1 treatment for 24 hrs. Magnified insets show direct sites of MG contact with surrounding cells. Scale bars = 10 µm. (**d**) Quantification of CTx-B in MG with and without N1 treatment for 24 hours. (**e**) Quantification of CTx-B intensity in area of direct MG contact with surrounding cells. (**f**) Ganglioside induced Cxcl10 secretion shown as a percentage of the PBS control (dotted line). *n* = 3. (**g**) Cxcl10 secretion from MG, MNLC and MNLC-MG co-cultures comparing N1 with acyl-N1 and expressed as percentage PBS control (dotted line). *n* = 3. (**h**) Prestoblue metabolism after MNLC-MG co-cultures were treated for 7 days with N1 or acyl-N1 shown as a percentage of PBS control (dotted line). *n* = 3. (**i**) Cxcl10 secretion from MG, MNLC and MNLC-MG co-cultures treated for 24 hours with N2 expressed as percentage PBS control (dotted line). *n* = 3. (**j**) Prestoblue metabolism of MG, MNLC and MNLC-MG co-cultures treated for 24 hours with N2 expressed as percentage PBS control (dotted line). *n* = 3. (**k**) Live imaging of MG morphology 7 days after addition of N2 to MG alone and MNLC-MG co-cultures. Scale bar = 20 µm. Graphs show the mean and s.e.m. of ‘*n*’ independent repeats. *p < 0.05, **p < 0.01, ***p < 0.001, # includes one repeat at the upper limit of detection for the assay.

To investigate how N1 may be mediating its effects in the MNLC-MG co-cultures we looked at cellular pathways previously found to be altered. N1 has been shown to be protective in the context of oxidative stress and, in neural stem cells, it down-regulated redox pathways^41^. Since mitochondria were previously found to be influenced by N1, cultures were stained with MitoSOX, mitochondrial superoxide probe. No changes in the intensity of MitoSOX (indicative of changes in superoxide production) were observed (Fig. 6a) and, as we previously observed using the MitoTracker probe (Fig. 1b), no changes in mitochondrial morphology were apparent. To confirm that there was no change in the redox balance of the cell a broader ROS probe, DCF, which disperses throughout the cell was also tested in both the co-cultures and the MG/MNLCs alone (Fig. 6b). This confirmed that no overt changes in the cellular redox environment were occurring.

### N1 changes MG GM1 at cell contact sites in MNLC-MG co-cultures

A further feature of N1 is its capacity to bind lipid membranes resulting in non-pathogenic changed membrane order^19,42,43^. A direct influence on the cell membranes may change how cells interact resulting in transduction of different signals. To look at cell membranes following N1 treatment of the co-cultures, MNLC with Cx3cr1-GFP MG were labelled with Alexa Fluor 647 conjugated cholera toxin subunit B (CTX-B), which binds to the ganglioside GM1, 24 hours after introduction of N1 to the culture. MG in the N1 treated co-cultures showed significantly greater CTX-B staining throughout the whole cell (Fig. 6c,d) and this was also found in the MG processes that contacted immediately surrounding cells (Fig. 6e). Gangliosides, including GM1 have been shown to change MG activation. To determine whether the increased GM1 staining within the culture may be directly involved in altering the co-culture Cxcl10 levels, MG, MNLCs and MG-MNLC co-cultures were incubated with 50 µg/ml mixed gangliosides (containing GM1) for 24 hours and media tested for cytokine levels. Cxcl10 was significantly increased in media harvested from the co-cultures (Fig. 6f). This suggests that N1 changes the MG membrane composition with the increased ganglioside content contributing to stimulation of Cxcl10 secretion.

### N1 induced changes in Cxcl10 secretion and metabolism required its charged domains

We additionally considered the importance of the biochemical properties of N1 itself in mediating the effects on the co-cultures. At physiological pH N1 is highly charged due to its two poly-basic regions. The charged regions of N1 may be important for driving the changes stimulated within the co-cultures. Therefore, sulfo-NHS Acetate was used to block the amine groups in these charged regions by irreversibly capping amines with an acyl group. The modified peptide is referred to as acyl-N1. Analysis of cytokine secretion when co-cultures were treated with 1 µM acyl-N1 (equimolar treatment to N1 alone), showed the influence of N1 on cytokine secretion was abolished at 24 hours (Fig. 6g) and the increase in metabolism seen after 7 days was also diminished (Fig. 6h). Accordingly, the charged regions of N1 appear important for its capacity to modulate the MNLC-MG co-culture cross-talk.

### N2 induces changes in Cxcl10 and metabolism of MG cultured alone

To consider whether the changes in Cxcl10 secretion and co-culture metabolism induced by N1 are specific to N1 function or may be induced by other N-terminal cleavages of PrP, we examined the N2 peptide. N2 is C-terminally truncated at the end of the octarepeat domain and therefore lacks the second charged region found in N1. The Cxcl10 secretion and metabolism of the MG, MNLC, and MNLC + MG cultures were measured 24 hours following addition of the N2. In contrast to the N1 results, no significant increases in either Cxcl10 secretion or metabolism in the MNLC + MG co-cultures were observed (Fig. 6i,j). However, the MG cultured alone showed an increase in both, suggesting a different functional engagement of N2. When the morphology of GFP-tagged MG were considered 7 days following treatment no morphological differences were observed in either the MG cultured alone or the MNLC-MG co-cultures (Fig. 6k). These results suggest that the N1 and N2 cleavage fragments may differ in their activities, influenced by the second charged region.

## Discussion

**Figure 7:**
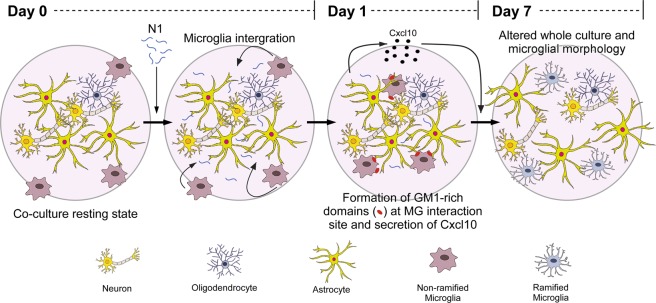
Schematic of the time course of N1 action. N1 stimulates changes in the membrane interactions between microglia and surrounding cells, increasing Cxcl10 secretion and causing changes in culture and microglial morphology.

The PrP N1 fragment has demonstrated neuroprotective activity in a number of cell systems. Since MG function to protect neurons in the brain we questioned whether N1 would be able to modulate MG functions. We further hypothesized that, as microglia do not exist in isolation within the brain, it might be important to consider the cross-talk between different cell types. The results presented herein suggest that N1 plays a protective maintenance role in communicating the needs of surrounding cells to MG. N1 modulated MNLC and MG metabolism, morphology and cytokine secretion only in the context of a co-culture system allowing direct cell-to-cell contact. The direct cell-to-cell connections were required for N1 to stimulate changes within the membrane environment where cell contacts were made. Our results are summarized graphically in Fig. 7 and listed in Table 1.

**Table 1:** Summary of changes induced by N1 and Cxcl10 within the culture systems. The conditions evaluated and observations are listed; those changed shown in bold. N/a – not applicable, n/d = not done.

	MNLC		MG		MNLC + MG		MNLC + MG (separated)	CXCL10
	24 h	7 d	24 h	7 d	24 h	7d	7d	7d
Metabolism	unchanged	unchanged	unchanged	unchanged	**Increased**	**Increased**	unchanged	**Increased**
MG morphology	n/a	n/a	Non-ramified	Non-ramified	Non-ramified	**Ramified**	Non-ramified	n/a
MNLC morphology	Less surface area / tightly packed clusters	Less surface area / tightly packed clusters	Less surface area / tightly packed clusters	Less surface area / tightly packed clusters	Less surface area / tightly packed clusters	**Increased surface area / less tightly packed clusters**	Less surface area / tightly packed clusters	**Increased surface area / less tightly packed clusters**
MG-MNLC contact	n/a	n/a	n/a	n/a	unchanged	**Increased**	n/a	n/a
Cxcl10 secretion	unchanged	unchanged	unchanged	unchanged	**Increased**	n/d	unchanged	n/a
GM1 contact site accumulation	n/a	n/a	n/a	n/a	**Increased**	n/d	n/a	n/a

The change in the MG morphology to a more ramified appearance in the co-cultures exposed to N1 indicated that MG activation was changed. Changes in MG activation state may result in changed phagocytic activity or altered cytokine secretion. Increased Cxcl10 secretion was observed in the co-cultures treated with N1 indicating the latter may be occurring. Cxcl10 is often considered an inflammatory cytokine, associated with ‘M1’ or pro-inflammatory microglia and neurotoxic outcomes. However, the ‘M1’ phenotype is also associated with heightened ROS production and increases in the secretion of other pro-inflammatory cytokines (such as TNFα)^2,6,7^. As both increased TNFα and ROS production were absent in our cultures, it is unlikely that N1 induces an ‘M1’ phenotype.

Basally in un-stressed mouse brain, Cxcl10 is produced by both astrocytes and MG^44^ (and to a lesser extent neurons^44,45^). As the Cxcl10 changes observed herein occurred in the shared environment of all these cells, we cannot conclude which cell type increased Cxcl10 production or whether increased secretion was stimulated for all types. The direct contact with MG may have induced astrocytes to secrete Cxcl10 that acts in an autocrine or paracrine fashion within the MNLCs. Such an effect would be consistent with signaling a protective response. While increased Cxcl10 is a marker for inflammation with negative outcomes, its secretion is stimulated during times of tissue damage as a functional response. Increased Cxcl10 is a signal to MG that they should migrate to the site of damage and correct it. Cxcr3 is the receptor for Cxcl10 (amongst other ligands). Mouse knock-out models of Cxcr3 show impaired recruitment of microglia and failure of microglia that do reach the site of damage to remove denervated distal dendrites after entorhinal cortex lesion^46^. In the culture environment used herein, without addition of a specific stressor, it is likely that the N1 mediated secretion of Cxcl10 caused, at least in part, the change in culture morphology, observed as increased surface area coverage of the cells. The Cxcl10 was feasibly a signal to improve the health of the culture and resulted in the increased metabolism that was observed. This assertion is supported by exogenously added Cxcl10 producing similar effects on MNLC in absence of MG, albeit within a relatively small concentration range. At higher concentrations the effect diminished rapidly, consistent with high Cxcl10 being a pro-inflammatory toxic mediator.

Previous studies have found that both N1 and N2 mediate cellular functions through modulating ROS and the mitochondria^21,41^. We hypothesized that N1 might alter these pathways in MG. If N1 changed MG ROS levels, possibly lowering them as seen previously, then this effect might be neuroprotective. However, no changes were found in mitochondrial or overall cellular ROS pathways. The divergence from previous studies most likely arises due to the different cell types or functions of the cell types and the different culture conditions or stresses. Within our unstressed, differentiated culture model the cells are expected to express different cell surface receptors that are specific to their functions. N1 may interact with different pathways depending upon the availability of various binding partners.

With ROS as a mediator of N1 function in the co-cultures negated, we directed our attention to other attributes of N1 that could be involved in its function. N1, and its shorter variant N2, have both been shown to interact with cell membrane lipids under diverse conditions, and these interactions were shown to change membrane ordering^19,42,43^. PrP has additionally been shown to directly bind the ganglioside, GM1^47^. The reported interaction was through the C-terminus and to the best of our knowledge the N-terminus has not been investigated in this context. Herein we found that N1 induced changes in the ganglioside GM1 at the sites of microglial interaction with surrounding cells. Gangliosides have been shown to activate microglia in culture. GM1 incompletely activates MG, with increases in expression of cyclooxygenase-2 but no change in nitric oxide production and TNFα secretion^48^. Furthermore, GM1 resides in cholesterol-rich domains. Disruption of cholesterol-rich domains using simvastatin reduces the migratory capacity of MG and down-regulates expression of Cxcr3^49^.

The charged domains of N1 influenced its efficacy, with both Cxcl10 secretion and metabolism unchanged when these regions were irreversibly blocked. The reliance upon the charged domains for stimulation of Cxcl10 secretion further supports that specific engagements at the membrane are critical for PrP functions, especially in the context of its N-terminal cleavage fragments. Various studies have previously found that these regions are important for binding to membrane components, including lipids and glycosaminoglycans^19,42,50–52^, and for internalization of full length PrP through engaging partners on the outer leaflet of the cell membrane^53–56^. N2 is much less charged than N1 due to the absence of the second charged domain in the N2 peptide. This reduction in charge could be the reason for the functional difference between N1 and N2, possibly by altering their ability to engage binding partners. The N2 fragment did not induce changes in the co-cultures, only in the MG on their own and, as MG will never exist alone in the brain, the lack of the second charged domain could render this peptide inactive in this functional context.

The proteases involved in the α-cleavage event, producing N1, are still a matter of debate^13,18,57–60^. This may in part be due to the α-cleavage site shifting depending on metallation of the protein or accessibility of the cleavage site; a minimum of three α-cut sites have been identified^60^. Many cell types display high basal levels of α-cleavage, suggesting it is an important functional/regulatory event^61^. Changes in protease activity or expression during damage or disease may shift production and secretion of N1, thus conceivably alerting MG of a need to migrate to and integrate at the site of injury mitigating the consequences. Potentially, changes in the C-terminus of N1 caused by the different α-cut sites may modulate the efficacy of such responses. The differences between the N1 and N2 peptides seen in the mono- and co-cultures appear to support this. Conversely, the ability to modulate cytokine secretion leads to the possibility of the system going awry. What is part of a delicate balance of production and response in a normal healthy environment could become an overactive driver of toxicity during disease. Both α- and β-cleavage events are increased in response to certain cellular stresses^20,62,63^ and during prion diseases PrP cleavage is shifted in favor of β-cleavage, producing N2. Astrocytes specifically were shown to have high levels of β-cleavage^64^. The shift toward β-cleavage is thought to be due to the mis-folding of PrP rendering the α-cleavage site unavailable. It also might have consequences for MG-mediated tissue homeostasis, with increased N2 and reduced N1 resulting in changed functional outcomes.

Overall, our results suggest that not only does N1 have neuromodulatory functions, as now seen in numerous systems, but also that these actions are acutely sensitive to context. The results presented herein highlight the complexity of brain tissue; neither the MNLCs nor MG on their own responded to N1 in the same way as when cultured together. This provides insight into how neuroprotective peptides within the brain can influence cellular cross-talk and how different brain cell types contact and communicate with each other. We cannot conclude that Cxcl10 secretion and GM1 changes are the only mediating factors in the observed metabolism and morphology changes; many more secretory factors or direct contacts at the membrane may also be participating. However, we can conclude that introduction of N1 into this system mediates direct changes within cell membranes resulting in greater cell contacts and changed secretion of Cxcl10, which exerts beneficial effects on neuronal lineage cells.

## Supplementary information

Supplementary Figure S1.

Supplementary Data S2.

Supplementary Figure S3.

Supplementary Data S4.

Supplementary Figure S5.

Supplementary Table S6.

## Data Availability

The datasets generated during and/or analysed during the current study are available from the corresponding author on reasonable request.
