# Supplementary material for: Prion protein N1 cleavage peptides stimulate microglial interaction with surrounding cells
Source: Sci Rep. 2020 Apr 20;10:6654. doi: 10.1038/s41598-020-63472-z (PMC7171115; doi:10.1038/s41598-020-63472-z)

***Supplementary Figure S1.*** *Microglia preparation.* Microglial preparations produce cultures with >99.9% purity. The below images show GFP-expressing microglia harvested from the Cx3cr1-GFP targeted mice and co-stained with DAPI to show all cellular nuclei. All DAPI staining cells are also expressing GFP indicating they are microglia.


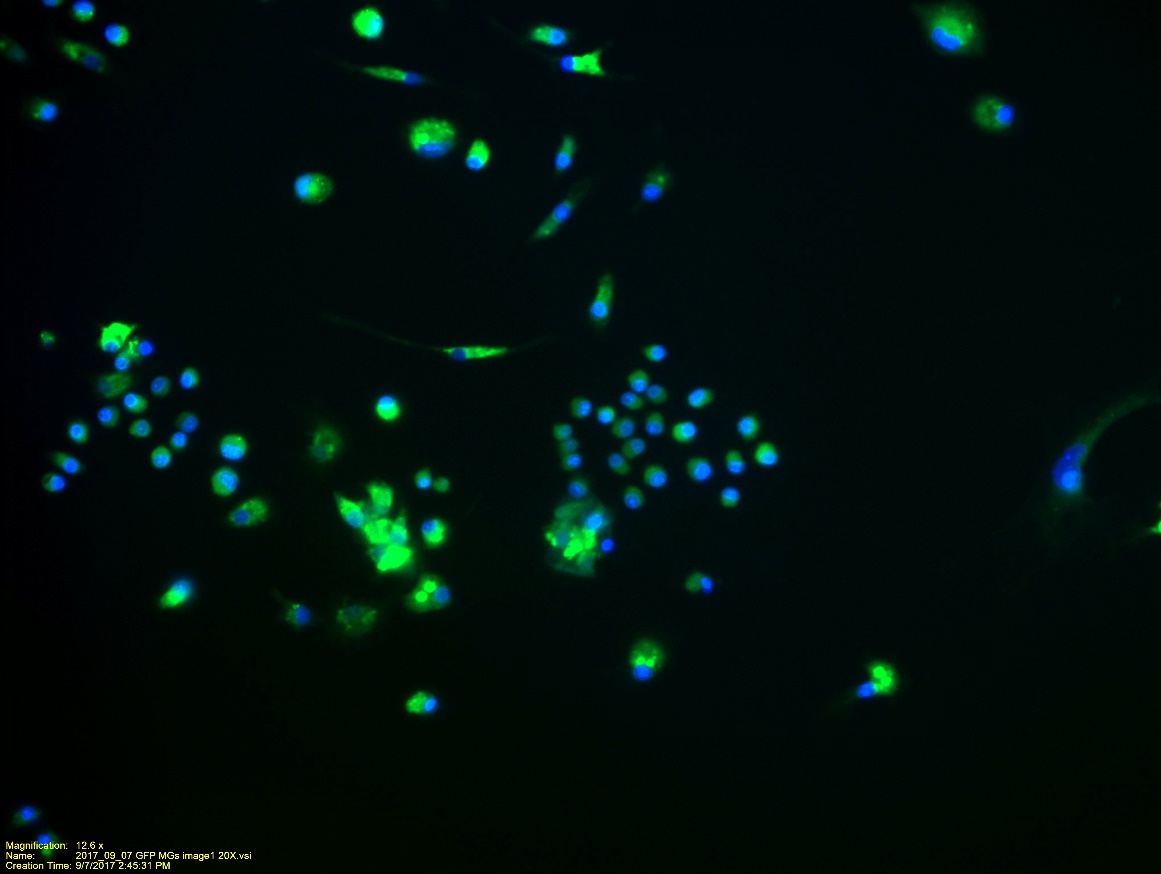

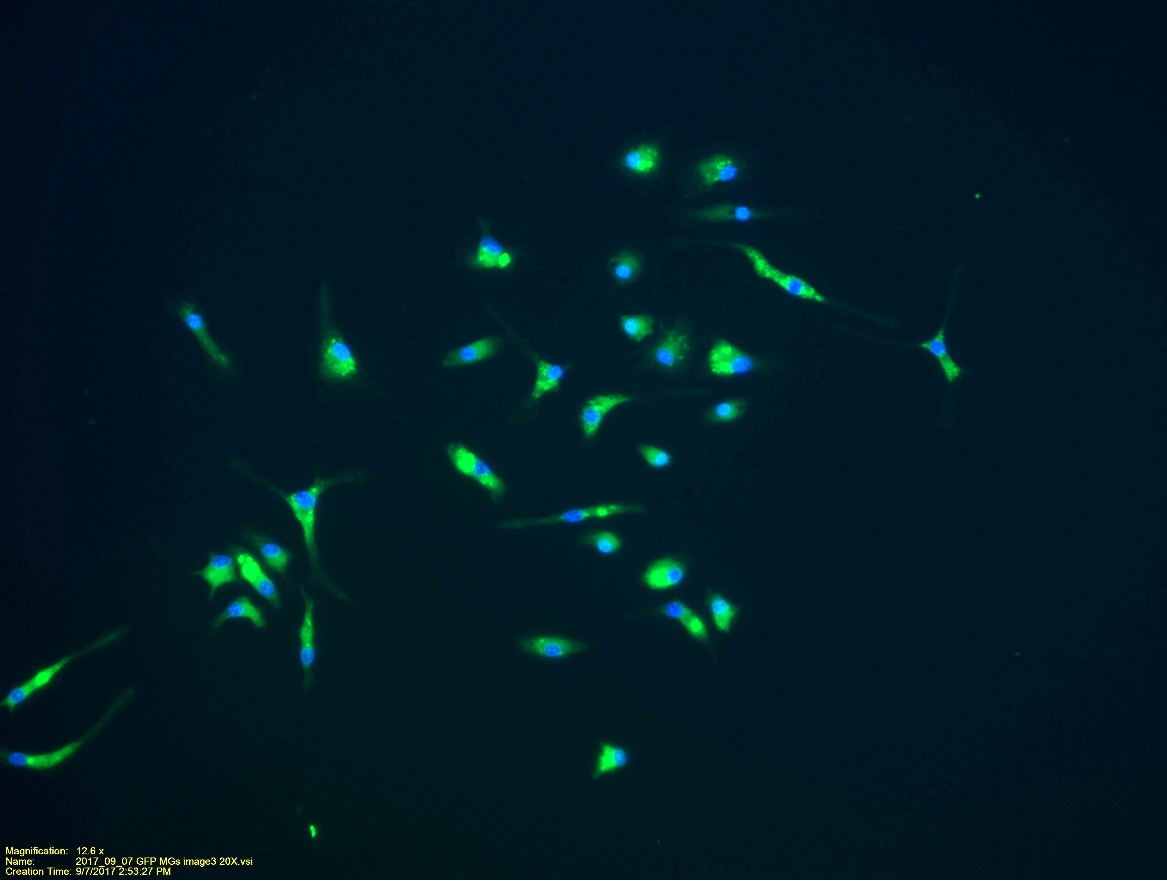

Supplement: Supplementary file 1 — Supplementary Figure S1. [file 41598_2020_63472_MOESM1_ESM.docx]
