# Supplementary material for: Prion protein N1 cleavage peptides stimulate microglial interaction with surrounding cells
Source: Sci Rep. 2020 Apr 20;10:6654. doi: 10.1038/s41598-020-63472-z (PMC7171115; doi:10.1038/s41598-020-63472-z)

Carroll, JA. Groveman, BR. Williams, K. Moore, R. Race, B. Haigh CL. **Prion protein N1 cleavage peptides stimulate microglial interaction with surrounding cells**

Supplementary Data S2. *Peptide QC data.* The high pressure liquid chromatography and mass spectrometry results for the peptide syntheses from Synpeptide are shown below.

CERTIFICATE OF ANALYSIS

**Product Name**	PrP_N1
**Lot No**	JT-68769
**Sequence**	NH2-KKRPKPGGWNTGGSRYPGQGSPGGNRYPPQGGTWG QPHGGGWGQPHGGSWGQPHGGSWGQPHGGGWGQGGG THNQWNKPSKPKTNLKHV - COOH
**Dissolution condition**	15%ACN+85%H2O
**Length**	89AA
**Modification**	N/A
**Molecular Weight (MW)**	9247.01
**Storage**	-20℃

**Test Items**	**Specifications**	**Results**
**Purity by HPLC**	95%	95.13%
**Peptide Content**	N/A	N/A
**Moisture content**	N/A	N/A
**Acetic acid content**	N/A	N/A
**Appearance**	White to off-white lyophilized powder	Conforms
**Quantity**	5-9mg	8.0mg

Certified by:
Quality Assurance Department

Date 10-25-2017

Note: this product is intended for research use only; not for diagnostic or human use.

Carroll, JA. Groveman, BR. Williams, K. Moore, R. Race, B. Haigh CL. **Prion protein N1 cleavage peptides stimulate microglial interaction with surrounding cells**

Sample Information

Order ID	: Syn-68769
Name	: PrP_N1
Sequence	: NH2-KKRPKPGGWNTGGSRYPGQGSPGGNRYPPQGGTWGQPHGG GWGQPHGGSWGQPHGGSWGQPHGGGWGQGGGTHNQWNKPSKP KTNLKHV - COOH
Lot No	: JT-68769
Pump A	: 0.1% Trifluoroacetic in 100% Water
Pump B	: 0.1% Trifluoroacetic in 100% Acetonrtrile
Total Flow	: 1ml/min
Wavelength	: 220nm
Analytial column type	: SHIMADZU Inertsil ODS-SP（4.6*250mm*5um）
Inj. Volume	: 30ul

Time	Module	Action	Value
0.00	Pumps	B.Conc	5
25.00	Pumps	B.Conc	65
25.01	Pumps	B.Conc	100
30.00	Pumps	B.Conc	100
30.01	Pumps	Stop	

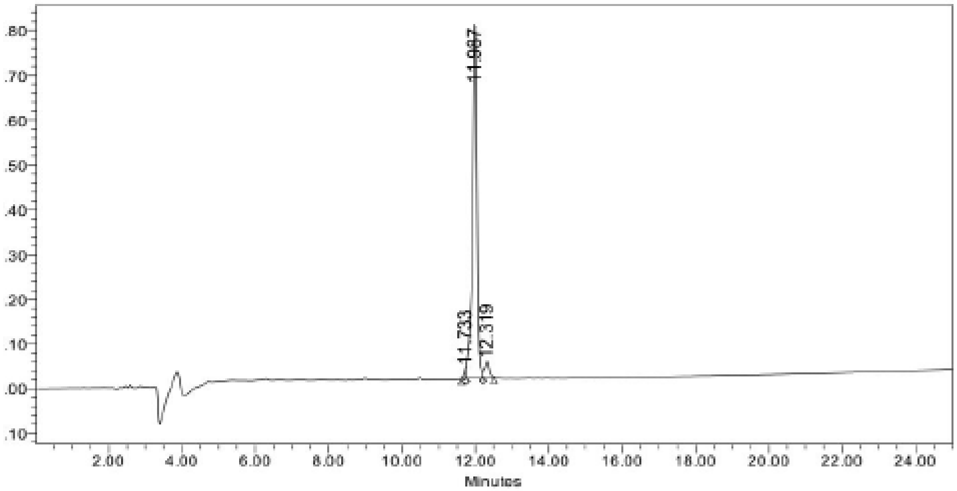

	RT	Area	% Area	Height
1	11.733	53678	0.78	22737
2	11.987	6580113	95.13	786337
3	12.319	283465	4.10	34583

Carroll, JA. Groveman, BR. Williams, K. Moore, R. Race, B. Haigh CL. **Prion protein N1 cleavage peptides stimulate microglial interaction with surrounding cells**

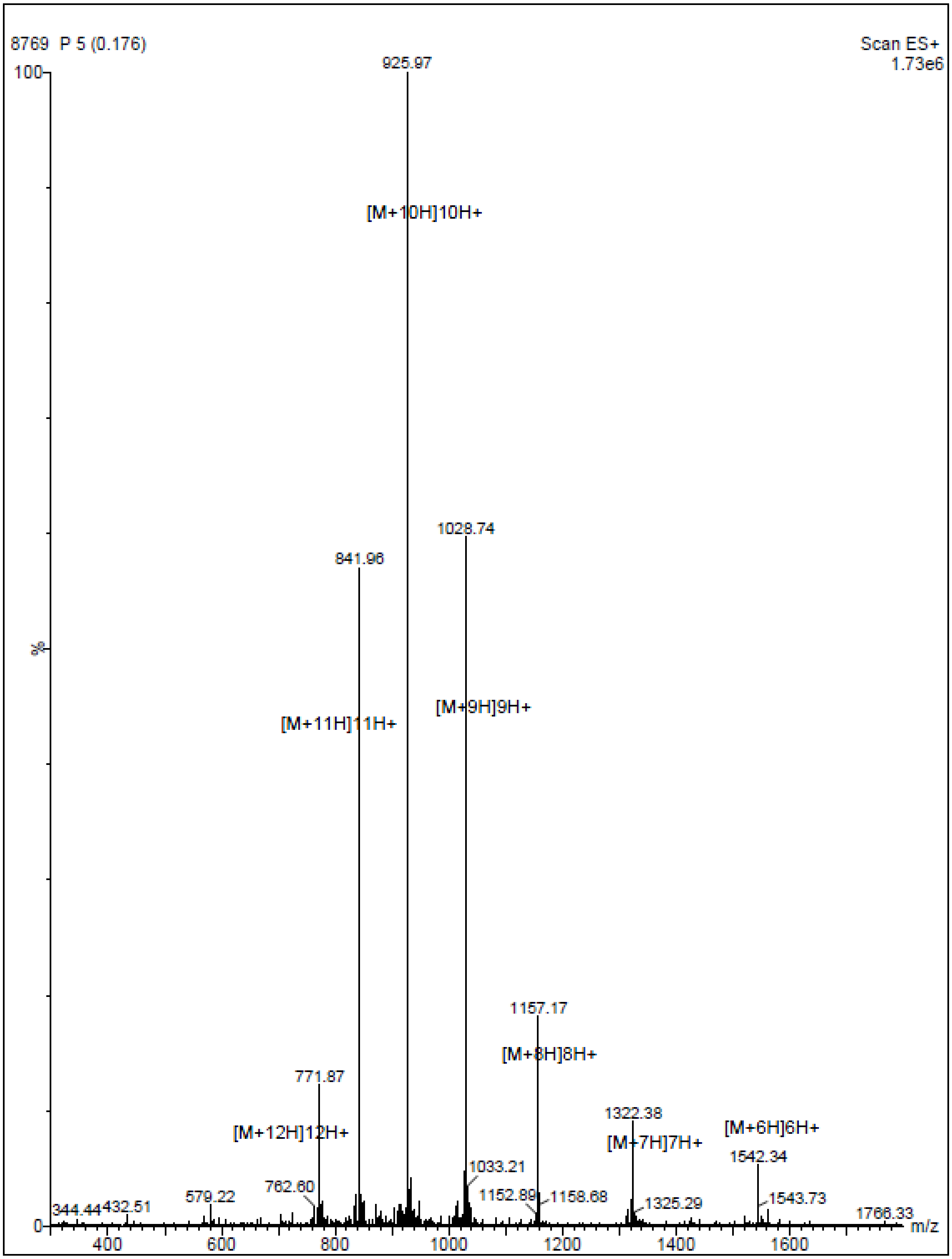

Carroll, JA. Groveman, BR. Williams, K. Moore, R. Race, B. Haigh CL. **Prion protein N1 cleavage peptides stimulate microglial interaction with surrounding cells**

CERTIFICATE OF ANALYSIS

**Product Name**	PrP_N2
**Lot No**	JT-81096
**Sequence**	NH2-KKRPKPGGWNTGGSRYPGQGSPGGNRYPPQGGTWGQ PHGGGWGQPHGGSWGQPHGGSWGQPHGGGWG- COOH
**Dissolution condition**	15%ACN+85%H2O
**Length**	67AA
**Modification**	N/A
**Molecular Weight (MW)**	6808.28
**Storage**	-20℃

**Test Items**	**Specifications**	**Results**
**Purity by HPLC**	95%	95.59%
**Peptide Content**	N/A	N/A
**Moisture content**	N/A	N/A
**Acetic acid content**	N/A	N/A
**Appearance**	White to off-white lyophilized powder	Conforms
**Quantity**	7mg(5-9mg)	7.0mg

Certified by:
Quality Assurance Department

Date 04/26/2019

Note: this product is intended for research use only; not for diagnostic or human use.

Carroll, JA. Groveman, BR. Williams, K. Moore, R. Race, B. Haigh CL. **Prion protein N1 cleavage peptides stimulate microglial interaction with surrounding cells**

Sample Information

Order ID	: Syn-81096
Name	: PrP_N2
Sequence	: NH2-KKRPKPGGWNTGGSRYPGQGSPGGNRYPPQGGTWGQPHGG GWGQPHGGSWGQPHGGSWGQPHGGGWG- COOH
Lot No	: JT-81096
Pump A	: 0.1% Trifluoroacetic in 100% Water
Pump B	: 0.1% Trifluoroacetic in 100% Acetonrtrile
Total Flow	: 1ml/min
Wavelength	: 220nm
Analytial column type	: SHIMADZU Inertsil ODS-SP（4.6*250mm*5um）
Inj. Volume	: 30ul

Time	Module	Action	Value
0.00	Pumps	B.Conc	5
25.00	Pumps	B.Conc	65
25.01	Pumps	B.Conc	100
30.00	Pumps	B.Conc	100
30.01	Pumps	Stop	

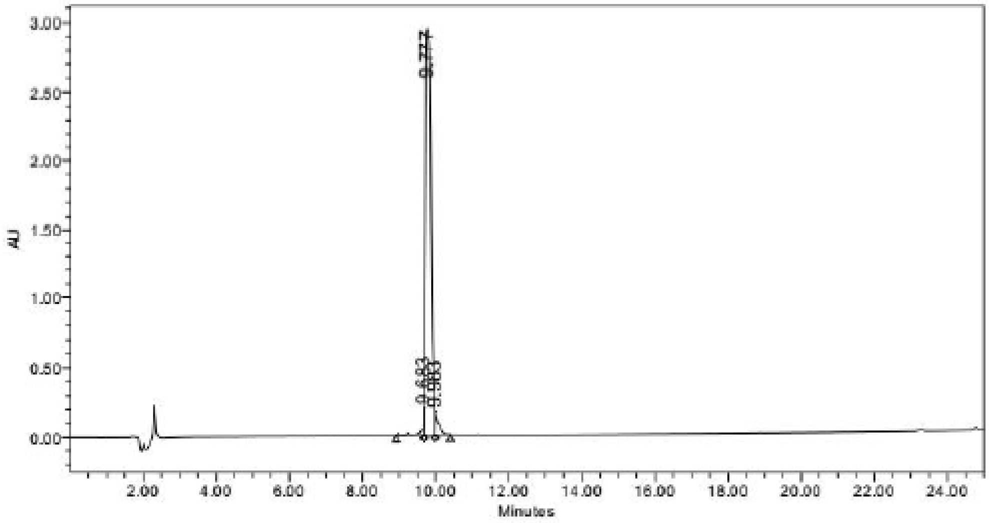

	RT	Area	% Area	Height
1	9.683	520923	1.54	196244
2	9.777	32238679	95.59	2970529
3	9.983	967058	2.87	167030

Carroll, JA. Groveman, BR. Williams, K. Moore, R. Race, B. Haigh CL. **Prion protein N1 cleavage peptides stimulate microglial interaction with surrounding cells**

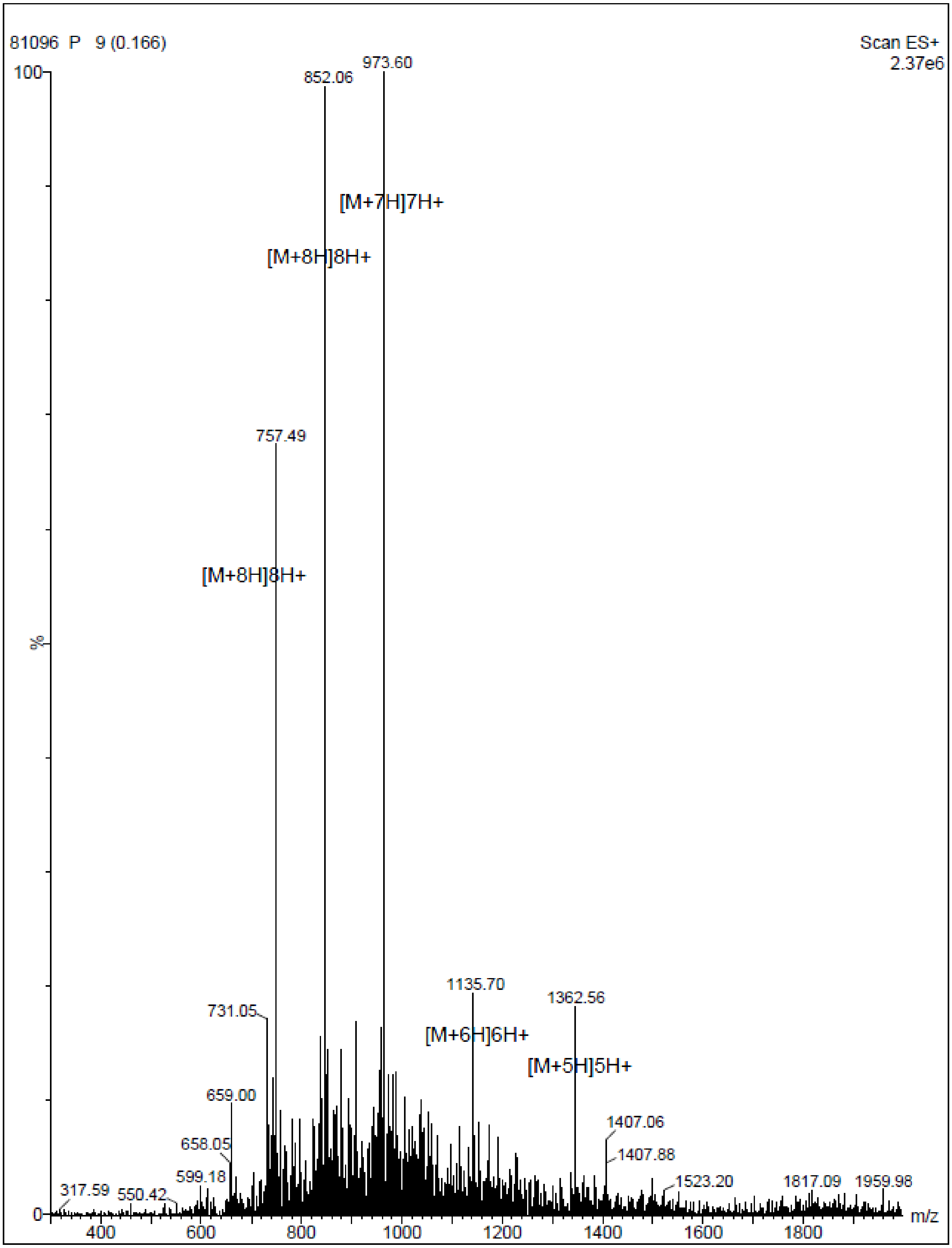

Supplement: Supplementary file 2 — Supplementary Data S2. [file 41598_2020_63472_MOESM2_ESM.pdf]
