# Supplementary material for: Prion protein N1 cleavage peptides stimulate microglial interaction with surrounding cells
Source: Sci Rep. 2020 Apr 20;10:6654. doi: 10.1038/s41598-020-63472-z (PMC7171115; doi:10.1038/s41598-020-63472-z)

***Supplementary Figure S3.*** *Mass spectrometry analysis of acyl-N1.* Treatment of the N1 peptide with sulfo-NHS acetate produced extensive acetylation within its N- and C-terminal charged regions, and, in the C-terminal region, no unmodified peptides were found. No modified lysine residues were observed in the un-reacted N1 peptide (data not shown). **A.** MS/MS spectrum representing one of the two doubly modified NKPSKPKTNL peptides observed in the C-terminal charged region. **B.** Corresponding fragmentation table for observed product ions and, **C.**, fragment ion error distribution. **D.** Extracted ion chromatogram (EIC) of m/z 605.8368. **E.** Sequence coverage of murine PrP with N1 indicated in pink. Also shown are the chymotrypsin cut sites (Chymo), the locations of the high scoring peptides and the sites with acetylated lysine residues (red boxed “a”). **F.** List of high-scoring peptides with -10lgP values ≥ 35.


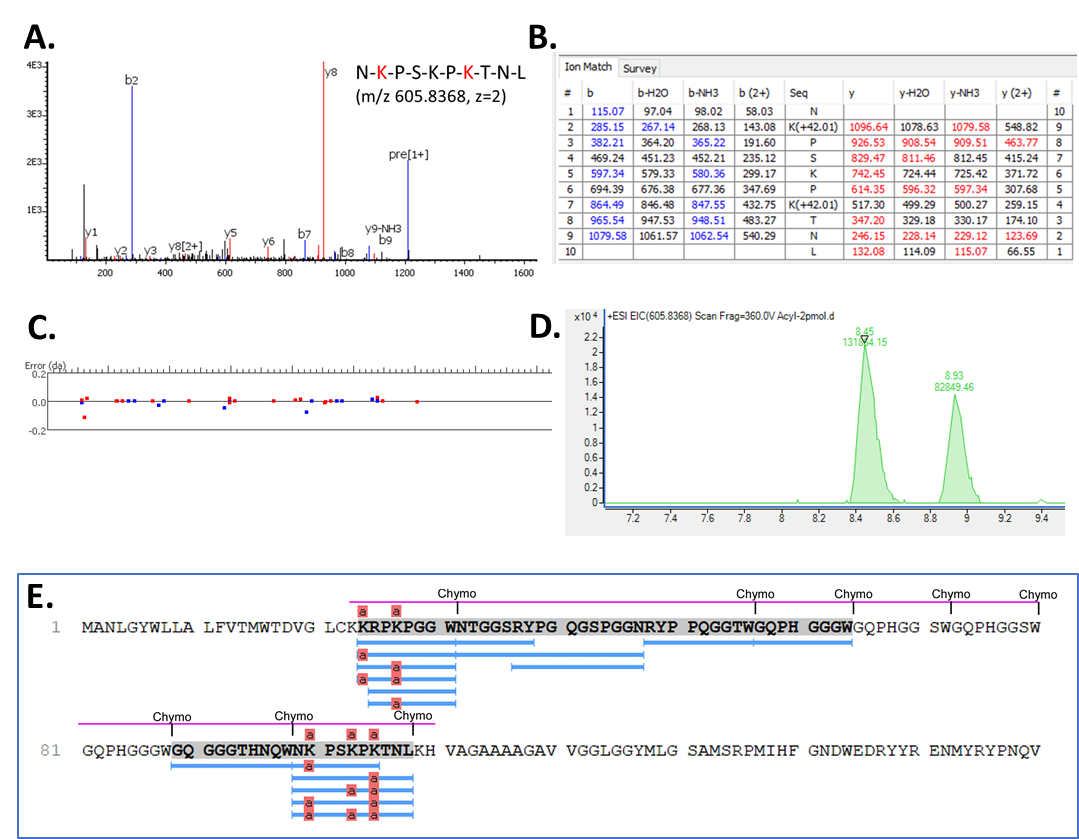


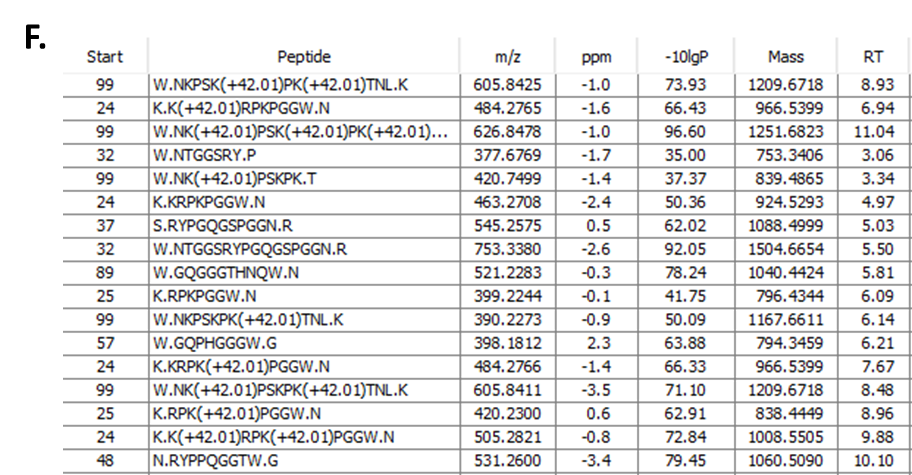

Supplement: Supplementary file 3 — Supplementary Figure S3. [file 41598_2020_63472_MOESM3_ESM.docx]
