# Supplementary material for: Prion protein N1 cleavage peptides stimulate microglial interaction with surrounding cells
Source: Sci Rep. 2020 Apr 20;10:6654. doi: 10.1038/s41598-020-63472-z (PMC7171115; doi:10.1038/s41598-020-63472-z)
Supplement: Supplementary file 4 — Supplementary Data S4. [file 41598_2020_63472_MOESM4_ESM.pdf]

Supplementary Data S4. *Individual data tables.* The data values included in each graph within the main text are shown below.

Figure 1b

	Prestoblue (% MNLC alone)							
	24 h				7 d			
Control	122.791	88.75334	94.20288	76.99891	63.06521	143.9	95.48732	65.88109
N1	94.5551	147.3266	278.2282	216.4537	225.4081	123.1417	136.42	197.7995

Figure 1d

	Area (%)					
	MNLC			MNLC + MG		
Control	4.332	6.064	5.134	10.995	18.705	14.493
N1	15.615	23.009	22.134	27.109	34.408	46.38

Figure 1f

	Non-ramified (%)			Ramified (%)		
Control	100	92.85714	84.21053	0	7.142857	15.78947
N1	47.61905	61.2069	28.57143	52.38095	38.79311	71.42857

Figure 1g

	% Microglia					
	MNLC associated			Unassociated		
Control	43.75	50	53.85	56.25	50	46.15
N1	80	80	63.63	20	20	36.37

Figure 2a

	Day		
	1	3	7
Prestoblue (% control MG)	122.7917	102.9982	71.21552
	110.4369	105.0112	100.343
	111.8537	99.9177	98.87364

Figure 2c

% Cells with beads

Control	N1	N2	T-a23
41.83168	53.76045	53.29815	43.44609
57.28155	50.87719	50.18916	33.05439
62.42038	60.60606	66.15385	46.21212

Figure 3a

	Ccl4 Concentration (pg/ml)							
	Control				N1			
MG	408.11	103.93	826.02	731.7	316.75	64.81	1077.21	1070.68
MNLC	23.71	82.46	77.87	87.05	33.39	33.39	44.79	54.5
MNLC + MG	1002.29	1559.46	1201.91	1919.54	1480.65	1701.41	1476.66	2375.07

Figure 3b

	Ccl24 Concentration (pg/ml)							
	Control				N1			
MG	131.01	269.14	145.6	174.86	733.72	490.015	184.37	193.78
MNLC	38.59	38.59	18.36	82.32	440.84	215.405	46.38	82.32
MNLC + MG	329.48	38.59	98.93	125.34	640.97	537.14	93.47	120.17

Figure 3c

	Ccl27 Concentration (pg/ml)							
	Control				N1			
MG	900.84	635.16	771.63	622.145	1024.43	1054.59	368.99	494.98
MNLC	407.85	410.07	354.77	458.71	130.64	67.11	268.88	327.07
MNLC + MG	1024.43	526.45	644.82	577.65	704.44	963.26	605.965	531.25

Figure 3d

	Cxcl10 Concentration (pg/ml)							
	Control				N1			
MG	454.81	377.39	227.6	227.6	368.42	292.92	189.45	198.09
MNLC	230.07	292.92	322.11	159.99	287.93	207.27	272.72	129.075
MNLC + MG	471.34	519.76	316.76	281.16	547.31	1059.95	938.63	783.03

Figure 4b

Cxcl10 Concentration (ng/ml)	% area covered 1	2	3	4
0	12.309	5.538	6.107	7.865
1	16.276	17.456	14.559	16.29
2.5	30.537	21.028	20.135	29.891
5	32.868	37.779	36.385	28.116
10	32.021	5.609	11.732	14.192
25	15.459	7.748	11.278	9.296
50	3.579	12.493	12.098	8.635

Figure 4c

Cxcl10 Concentration (ng/ml)	Total pixel intensity					
	NF-L			GFAP		
0	2.61E+09	2.49E+09	3.15E+09	1.33E+09	1.44E+09	3.13E+09
1	3.21E+09	2.58E+09	4.76E+09	2.06E+09	5.19E+09	2.68E+09
2.5	3.61E+09	3.12E+09	3.43E+09	3.14E+09	2.18E+09	3.41E+09
5	4.11E+09	5.29E+09	4.56E+09	5.13E+09	4.95E+09	4.61E+09
10	1.6E+09	3.39E+09	5.14E+09	2.76E+09	1.7E+09	4.3E+09
25	2.8E+09	2.73E+09	1.95E+09	1.85E+09	2.07E+09	1.49E+09
50	3.67E+09	3.06E+09	1.38E+09	1.63E+09	5.75E+08	1.45E+09

Figure 4d

Cxcl10 Concentration (ng/ml)	Prestoblue metabolism (% control)			
0	100	100	100	100
1	119.3377	123.8034	112.6949	89.7509
2.5	200.5704	216.0687	223.6279	133.76
5	406.8875	392.0374	382.863	178.2631
10	73.0861	74.6608	89.7742	136.9548
25	100.5637	106.5888	125.0236	144.6124
50	90.9269	80.8373	8.4503	118.5219

Figure 4f

Prestoblue metabolism (% control)

Control		Cxcr3 neutralising antibody	
Control	N1	Control	N1
100	132.9394	84.30282	87.58554
100	157.3753	78.05907	25.03516
100	151.4279	80.25446	106.6484

Figure 5c

	Prestoblue metabolism (% control)					
	MG			MNLC		
Co-exposed	111.1959	79.60322	99.26368	128.6904	79.61492	154.4061
MNLC primed	86.0667	61.36907	111.7076	101.4503	86.55151	81.15023

Figure 5d

Cxcl10 (% PBS control)

Co-culture	Co-exposed (A)	MNLC primed (B)
233.6431	53.35285	113.9806
206.9007	40.68697	156.9126
172.6021	101.16	98.39599
131.2212	107.2243	103.3215

Figure 6b

	DCF production (FU/s)								
	MG			MNLC			MNLC + MG		
Control	0.14	0.1	0.14	0.26	0.23	0.21	0.16	0.16	0.19
N1	0.11	0.13	0.11	0.21	0.14	0.24	0.14	0.14	0.16

Figure 6d

Whole cell mean pixel intensity (FU)	
PBS	N1
2870.027	4415.197
3316.713	4571.388
3309.222	5433.314

Figure 6e

MG-cell contact site mean pixel intensity (FU)	
PBS	N1
3115.159	5102.973
3329.5	4205.074
1957.29	3827.803

Figure 6f (*upper limit of assay detection reached)

Ganglioside Induced Cxcl10 (% vehicle control)		
MG	MNLC	MNLC + MG
80.77633	116.1092	233.1985
78.43948	99.43803	153.4229
87.67496	70.9541	343.5109*

Figure 6g

	Cxcl10 (%PBS control)					
	N1			Acyl-N1		
MG	114.4695	58.86282	58.86282	105.3723	81.55118	89.18653
MNLC	117.0392	85.26669	88.95097	66.82848	133.0564	121.1944
MNLC + MG	206.9007	172.6021	161.8357	88.99655	94.14203	23.37041

Figure 6h

Prestoblue metabolism (% control)

N1	N1-sulfo-NHS
197.7995	143.2817
174.2749	88.6153
153.5562	132.3992

Figure 6i

N2 Induced Cxcl10 (% control)

MG	MNLC	MNLC + MG
151.5178	191.6049	112.2653
132.7264	88.89388	72.50525
146.4202	90.77234	124.5249

Figure 6j

N2 Prestoblue metabolism (% control)

MG	MNLC	MNLC + MG
149.7831	90.12155	127.9611
159.1088	95.90614	97.0698
119.2196	99.63388	104.2989
