# Supplementary material for: Prion protein N1 cleavage peptides stimulate microglial interaction with surrounding cells
Source: Sci Rep. 2020 Apr 20;10:6654. doi: 10.1038/s41598-020-63472-z (PMC7171115; doi:10.1038/s41598-020-63472-z)

***Supplementary Figure S5.*** *NF-L and background staining.* **A.** Example NF-L images showing MG are mostly isolated from neurons. Some cross-reactivity of the NF-L antibody with non-neuronal cells is also present as shown by background staining in cells lacking neuronal morphology. **B.** Control images showing the tissue background under the image collection conditions used for GFP imaging and background staining of the AlexaFluor-488 and -647 secondary antibodies used for immunofluorescence.


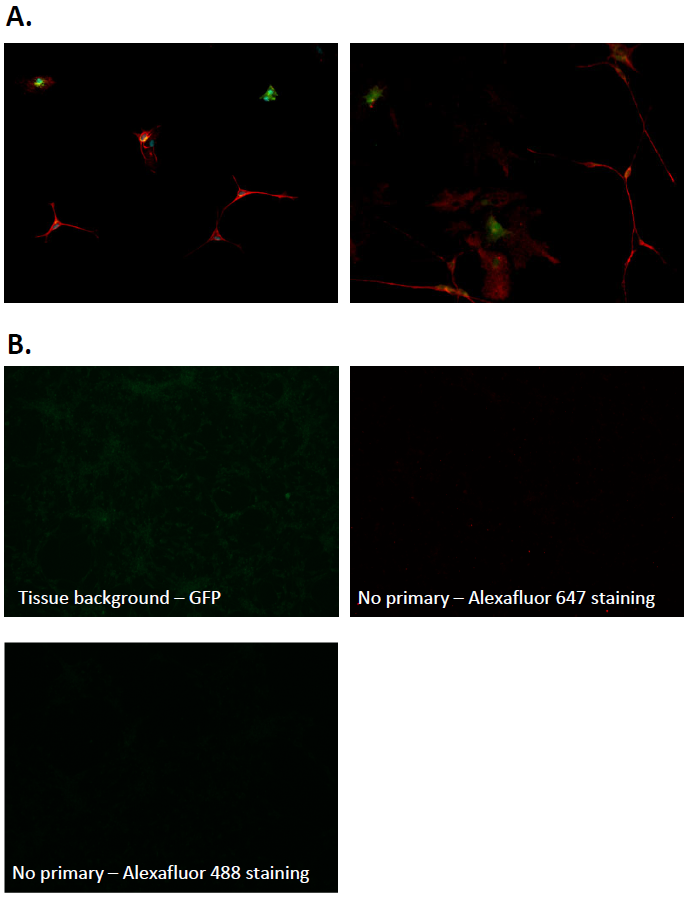

Supplement: Supplementary file 5 — Supplementary Figure S5. [file 41598_2020_63472_MOESM5_ESM.docx]
