# Supplementary material for: Prion protein N1 cleavage peptides stimulate microglial interaction with surrounding cells
Source: Sci Rep. 2020 Apr 20;10:6654. doi: 10.1038/s41598-020-63472-z (PMC7171115; doi:10.1038/s41598-020-63472-z)
Supplement: Supplementary file 6 — Supplementary Table S6. [file 41598_2020_63472_MOESM6_ESM.pdf]

Supplementary Table S6. *Complete cytokine panel data for N1 treatments.* Mean concentration (+/- standard deviation) in pg/ml and fold change (+/- standard deviation) for each cytokine analysed following treatment of MG, MNLC or MG-MNLC co-cultures with PBS (control) or N1. *n* = 4. Values determined significant by two-way ANOVA analysis (corresponding to the graphs in Figure 3) are highlighted in red. ND = not detected.

	Co-cultures			MNLCs			MG cells		
Cytokine	Control	N1	Fold change	Control	N1	Fold change	Control	N1	Fold change
BCA-1/CXCL13	713.76 (+/-173.02)	607.38 (+/-182.44)	0.84 (+/-0.12)	336.90 (+/-145.85)	286.63 (+/-86.25)	1.28 (+/-1.39)	755.4 (+/-193.40)	662.4 (+/-239.88)	0.86 (+/-0.12)
CTACK/CCL27	693.34 (+/-225.99)	682.18 (+/-182.54)	1.0 (+/-0.29)	407.85 (+/-52.01)	198.43 (+/-120.21)	0.54 (+/-0.24)	710.38 (+/-127.69)	687.59 (+/-326.53)	0.97 (+/-0.45)
ENA-78/CXCL5	47.98 (+/-21.53)	42.01 (+/-23.50)	0.84 (+/-0.17)	30.43 (+/-21.39)	ND	ND	66.25 (+/-28.20)	48.80 (+/-35.77)	0.58 (+/-0.41)
EOTAXIN/CCL11	8.34 (+/-1.18)	8.23 (+/-2.13)	0.98 (+/-0.21)	4.81 (+/-0.64)	4.08 (+/-1.64)	0.83 (+/-0.21)	9.55 (+/-3.86)	8.40 (+/-5.73)	0.84 (+/-0.23)
EOTAXIN-2/CCL24	148.09 (+/-126.26)	385.78 (+/-279.44)	3.97 (+/-4.37)	44.47 (+/-26.98)	200.07 (+/-165.96)	5.68 (+/-4.80)	180.15 (+/-62.06)	418.38 (+/-279.50)	2.65 (+/-2.08)
FRACTALKINE/CX3CL1	41.05 (+/-4.57)	38.89 (+/-8.07)	0.97 (+/-0.3063)	30.94 (+/-6.21)	25.78 (+/-6.91)	0.83 (+/-0.15)	41.63 (+/-9.65)	30.16 (+/-22.43)	0.68 (+/-0.33)
GM-CSF	5.67 (+/-2.34)	5.59 (+/-1.44)	1.25 (+/-0.79)	4.52 (+/-1.92)	3.84 (+/-2.12)	1.14 (+/-1.07)	6.33 (+/-2.79)	5.27 (+/-1.65)	0.92 (+/-0.34)
I-309/CCL1	1.72 (+/-0.24963974	1.57 (+/-0.33)	0.95 (+/-0.08)	0.93 (+/-0.18)	0.77 (+/-0.25)	0.94 (+/-0.30)	1.84 (+/-0.33)	1.52 (+/-0.70)	0.81 (+/-0.25)
INF-g	4.07 (+/-1.16)	3.07 (+/-0.75)	0.79 (+/-0.28)	2.38 (+/-0.51)	1.83 (+/-0.39)	0.80 (+/-0.26)	4.74 (+/-0.56)	3.6 (+/-1.13)	0.76 (+/-0.24)
IL-1b	24.49 (+/-1.60)	19.31 (+/-2.40)	0.28 (+/-0.48)	ND	ND	ND	23.61 (+/-9.10)	40.57 (+/-11.43)	0.73 (+/-0.96)
IL-2	6.00 (+/-5.29)	4.23 (+/-3.36)	0.81 (+/-0.19)	2.41 (+/-2.09)	0.69 (+/-0.34)	0.61 (+/-0.58)	6.57 (+/-5.86)	4.46 (+/-4.04)	0.74 (+/-0.25)
IL-4	0.81 (+/-0.26)	0.78 (+/-0.10)	1.02 (+/-0.28)	0.30 (+/-0.15)	0.30 (+/-0.11)	1.19 (+/-0.82)	0.93 (+/-0.23)	0.90 (+/-0.47)	0.91 (+/-0.29)
IL-6	7.76 (+/-1.57)	8.81 (+/-3.07)	1.13 (+/-0.32)	4.22 (+/-0.74)	2.43 (+/-1.40)	0.58 (+/-0.31)	12.02 (+/-5.44)	9.33 (+/-8.00)	0.69 (+/-0.26)
IL-10	61.28 (+/-22.52)	27.45 (+/-11.68)	0.63 (+/-0.62)	41.87 (+/-17.50)	ND	ND	69.52 (+/-8.48)	34.11 (+/-39.03)	0.44 (+/-0.67)
IL-16	2.23 (+/-0.43)	2.04 (+/-0.34)	0.96 (+/-0.27)	1.06 (+/-0.53)	0.88 (+/-0.32)	1.72 (+/-2.26)	2.81 (+/-0.36)	2.23 (+/-0.91)	0.78 (+/-0.28)
IP-10/CXCL10	397.26 (+/-116.12)	832.23 (+/-221.19)	2.24 (+/-0.82)	233.02 (+/-74.61)	205.21 (+/-75.89)	0.88 (+/-0.21)	321.85 (+/-113.33)	262.22 (+/-84.91)	0.82 (+/-0.04)
I-TAC/CXCL11	151.61 (+/-48.25)	122.59 (+/-29.92)	0.83 (+/-0.17)	96.33 (+/-31.72)	67.47 (+/-37.07)	0.66 (+/-0.20)	185.89 (+/-82.80)	131.61 (+/-105.49)	0.65 (+/-0.20)
KC/CXCL1	29.38 (+/-22.94)	56.22 (+/-51.87)	1.75 (+/-0.69)	6.72 (+/-6.13)	11.48 (+/-7.29)	1.31 (+/-0.39)	20.51 (+/-17.04)	23.65 (+/-29.85)	0.90 (+/-0.42)
MCP-1/CCL2	400.80 (+/-137.39)	409.49 (+/-192.46)	1.08 (+/-0.37)	164.07 (+/-71.47)	110.46 (+/-23.61)	0.57 (+/-0.09)	968.00 (+/-1817.04)	62.80 (+/-35.40)	0.22 (+/-0.24)
MCP-3/CCL7	48.56 (+/-29.42)	100.03 (+/-65.50)	2.44 (+/-2.11)	21.23 (+/-27.29)	19.85 (+/-14.21)	1.54 (+/-1.03)	108.75 (+/-236.01)	158.71 (+/-347.71)	1.08 (+/-0.34)
MCP-5/CCL12	6.71 (+/-2.58)	12.34 (+/-8.59)	1.78 (+/-1.05)	2.56 (+/-1.02)	1.63 (+/-0.18)	0.76 (+/-0.27)	9.88 (+/-13.04)	22.66 (+/-40.77)	1.39 (+/-0.88)
MDC/CCL22	5.85 (+/-1.44)	5.27 (+/-1.65)	0.92 (+/-0.09)	2.81 (+/-0.81)	1.47 (+/-0.96)	0.45 (+/-0.18)	7.05 (+/-1.80)	7.22 (+/-5.34)	0.96 (+/-0.45)
MIP-1a/CCL3	734.32 (+/-590.91)	841.52 (+/-636.85)	1.22 (+/-0.17)	1.89 (+/-1.07)	1.06 (+/-0.26)	0.68 (+/-0.35)	1133.29 (+/-1058.27)	1266.48 (+/-1311.50)	1.06 (+/-0.24)
MIP-1b/CCL4	1420.8 (+/-404.57)	1758.45 (+/-424.28)	1.26 (+/-0.16)	64.74 (+/-28.00)	41.52 (+/-10.19)	0.75 (+/-0.45)	1185.79 (+/-1521.34)	1384.19 (+/-1740.44)	1.06 (+/-0.35)
MIP-2/CXCL2	99.956 (+/-51.24)	131.69 (+/-73.43)	1.30 (+/-0.48)	11.14 (+/-2.39)	7.36 (+/-1.347156264	0.70 (+/-0.26)	228.23 (+/-124.52)	239.63 (+/-140.47)	1.05 (+/-0.18)
MIP-3a/CCL20	23.46 (+/-10.29)	14.33 (+/-2.41)	0.69 (+/-0.02)	22.45 (+/-4.34)	15.05 (+/-3.13)	0.71 (+/-0.13)	30.74 (+/-7.58)	23.99 (+/-7.77)	0.56 (+/-0.54)
MIP-3b/CCL19	44.96 (+/-18.97)	40.68 (+/-20.81)	0.87 (+/-0.15)	28.10 (+/-10.23)	10.38 (+/-3.36)	0.45 (+/-0.27)	55.36 (+/-26.64)	45.97 (+/-39.27)	0.79 (+/-0.32)
RANTES/CCL5	34.30 (+/-34.29)	37.53 (+/-35.03)	1.24 (+/-0.53)	3.61 (+/-1.60)	2.55 (+/-1.64)	0.84 (+/-0.75)	25.08 (+/-17.47)	31.28 (+/-27.72)	1.15 (+/-0.38)
SCYB16/CXCL16	26.01 (+/-12.94)	30.88 (+/-14.22)	1.25 (+/-0.20)	1.37 (+/-0.37)	0.88 (+/-0.05)	0.69 (+/-0.24)	40.52 (+/-46.64)	43.05 (+/-45.22)	1.15 (+/-0.14)
SDF-1a/CXCL12	59.92 (+/-6.47)	50.74 (+/-15.68)	0.76 (+/-0.27)	28.47 (+/-11.49)	20.99 (+/-3.12)	0.62 (+/-0.18)	71.91 (+/-24.70)	70.29 (+/-38.86)	0.54 (+/-0.50)
TARC/CCL17	21.82 (+/-6.31)	20.42 (+/-5.52)	0.95 (+/-0.21)	17.21 (+/-6.47)	13.21 (+/-4.06)	0.81 (+/-0.25)	26.206 (+/-4.52)	21.13 (+/-11.09)	0.80 (+/-0.39)
TECK/CCL25	147.16 (+/- 0)	124.88 (+/-10.73)	0.85 (+/-0.07)	89.15 (+/-5.93)	57.89 (+/-13.46)	0.65 (+/-0.11)	164.06 (+/-43.09)	167.03 (+/-17.29)	0.57 (+/-0.50)
TNF-a	13.61 (+/-2.54)	6.13 (+/-3.03)	0.44 (+/-0.29)	10.78 (+/-2.95)	1.43 (+/-1.93)	0.13 (+/-0.17)	16.54 (+/-1.58)	8.07 (+/-7.29)	0.50 (+/-0.48)
